# Natural Phaeosphaeride A Derivatives Overcome Drug Resistance of Tumor Cells and Modulate Signaling Pathways

**DOI:** 10.3390/ph15040395

**Published:** 2022-03-24

**Authors:** Victoria Abzianidze, Natalia Moiseeva, Diana Suponina, Sofya Zakharenkova, Nadezhda Rogovskaya, Lidia Laletina, Alvin A. Holder, Denis Krivorotov, Alexander Bogachenkov, Alexander Garabadzhiu, Anton Ukolov, Vyacheslav Kosorukov

**Affiliations:** 1 Research Institute of Hygiene, Occupational Pathology and Human Ecology, Federal Medical Biological Agency, p/o Kuz’molovsky, 188663 Saint Petersburg, Russia; dina.lykina.97@mail.ru (D.S.); sofya.zakharenkova@gmail.com (S.Z.); nadin-r@mail.ru (N.R.); denhome@bk.ru (D.K.); alexterve@gmail.com (A.B.); antonukolov@gmail.com (A.U.); 2 Laboratory of Tumor Cell Genetics, Institute of Carcinogenesis, N.N. Blokhin National Medical Research Center of Oncology of the Ministry of Health of the Russian Federation, 115478 Moscow, Russia; n.i.moiseeva@gmail.com (N.M.); panlidia@gmail.com (L.L.); 3 Department of Chemistry and Biochemistry, Old Dominion University, 4501 Elkhorn Avenue, Norfolk, VA 23529, USA; 4 Saint Petersburg State Technological Institute (Technical University), 190013 Saint Petersburg, Russia; gar-54@mail.ru; 5 Laboratory of Transgenic Drugs, N.N. Blokhin National Medical Research Center of Oncology of the Ministry of Health of the Russian Federation, 115478 Moscow, Russia; atgtga@mail.ru

**Keywords:** natural phaeosphaeride A, anti-tumor activity, oxidative stress, multidrug resistance, P-glycoprotein, signaling pathways, JNK, ERK 1/2

## Abstract

In the present study, natural phaeosphaeride A (PPA) derivatives are synthesized. Anti-tumor studies are carried out on the PC3, K562, HCT-116, THP-1, MCF-7, A549, NCI-H929, Jurkat, and RPMI8226 tumor cell lines, and on the human embryonic kidney (HEK293) cell line. All the compounds synthesized turned out to have better efficacy than PPA towards the tumor cell lines listed. Among them, three compounds exhibited an ability to overcome the drug resistance of tumor cells associated with the overexpression of the P-glycoprotein by modulating the work of this transporter. Luminex xMAP technology was used to assess the effect of five synthesized compounds on the activation of intracellular kinase cascades in A431 cells. MILLIPLEX MAP Multi-Pathway Magnetic Bead 9-Plex was used, which allowed for the simultaneous detection of the following nine phosphorylated protein markers of the main intracellular signaling pathways: a universal transcription factor that controls the expression of immune-response genes, apoptosis and cell cycle NFκB (pS536); cAMP-dependent transcription factor (CREB (pS133); mitogen-activated kinase p38 (pT180/pY182); stress-activated protein kinase JNK (pT183/pY185); ribosomal SK; transcription factors STAT3 (pS727) and STAT5A/B (pY694/699); protein kinase B (Akt) (pS473); and kinase regulated by extracellular signals ERK1/2 (pT185/pY187). The effect of various concentrations of PPA derivatives on the cell culture was studied using xCelligence RTCA equipment. The compounds were found to modulate JNK, ERK1/2, and p38 signaling pathways. The set of activated kinase cascades suggests that oxidative stress is the main probable mechanism of the toxic action of PPA derivatives.

## 1. Introduction

Multiple drug resistance (MDR) of tumor cells is a major obstacle for the chemotherapy of malignant neoplasms. MDR is a system for protecting cells simultaneously from many drugs that differ in their chemical structure and mechanism of action on the cells [1]. Among the known molecular causes of drug resistance in human tumors is the currently well-characterized mechanism of the increased activity of the ABC family protein (ATP-binding cassette) P-glycoprotein (ABCB1, hereafter P-gp), encoded by the *ABCB1 (MDR1)* gene [2].

One of the characteristic features of P-gp is its broad substrate specificity, ranging from small molecules, such as organic cations, carbohydrates, amino acids, and some antibiotics, to macromolecules, such as polysaccharides and proteins [3]. Usually, P-gp substrates are weakly amphipathic or relatively hydrophobic, and most of them contain aromatic rings and a positively charged tertiary nitrogen atom in their structure [3].

Basal expression levels of ABC transporters in tumors vary significantly, depending on the cell type. Thus, tumors of the intestine and ureters, and hepatocellular carcinoma, are characterized by a high constitutive expression of P-gp and (or) the *ABCB1* gene [4]. However, it has become clear from clinical practice that the basal level of *ABCB1* gene expression may not always be a diagnostic sign of tumor resistance. In acute myelocytic leukemia, small cell lung cancer, and in breast and ovarian tumors, the initial level of ABCB1 expression is usually low. However, after chemotherapy, an increase in P-gp expression has been shown and the appearance of resistance has been observed. Thus, P-gp expression was found in 14% of patients with breast tumors during the initial examination, while, after chemotherapy, it increased up to 43% [5].

P-gp exports a wide range of structurally and mechanically unrelated chemotherapeutic agents, covering almost the whole spectrum of anticancer drugs approved for clinical use, including vinca alkaloids, podophyllotoxins, taxanes, tyrosine kinase inhibitors, camptothecin analogs, antibiotics, anthracyclines, antimetlotoxins and epiodofenes, and anthrodophylenes. Therefore, over the past several decades, numerous attempts have been made to solve the problem of MDR caused by proteins of the ABC transporter family using inhibitors of these proteins [6]. Inhibitors can modulate P-gp function by breaking ATP hydrolysis, changing P-gp expression, or by reversible/irreversible competition for the binding site. One of the most common mechanisms demonstrated by classical P-gp inhibitors is the competition with drug binding sites.

To date, there are three generations of P-gp inhibitors, some of which have been tested in clinical trials, but unfortunately most have failed [7] due to the nonspecific toxicity of the inhibitor, an increase in the toxicity of the main drug, and an increase in the toxicity of a co-administered drug in healthy tissues by inhibiting the basic activity of P-gp. Hence, there is an urgent need to identify new, more effective, and non-toxic P-gp inhibitors without drug interactions [8].

Recently, compounds obtained from natural products were viewed as promising ABC transporter modulators for MDR control. Flavonoids and terpenoids have been widely studied as mono- or double modulators of transporter proteins [9].

Phaeosphaeride A (PPA) is a natural product that possesses anti-tumor properties. PPA was first isolated from the endophytic fungus FA39 (Phaeosphaeria avenaria) by Clardi et al. in 2006, and was reported to inhibit STAT3/DNA binding with an IC50 value of 0.61 mM [3]. In this case, PPA was chosen for its ability to inhibit the signaling pathway of the signal transducer and transcription activator 3 (STAT3). The stereochemical configuration of this natural product was established by the complete synthesis of ent-phaeosphaeride A and PPA [10] and by using X-ray diffraction [11]. Aberrant activity of the STAT3 protein was found in many types of tumors, including breast, ovarian, kidney, prostate, and lung cancers, as well as in some leukemias and multiple myeloma [12,13]. STAT3 promotes oncogenesis by regulating the expression of various target genes, including cell cycle regulators, angiogenic factors, and anti-apoptotic genes [12,14,15,16,17]. Various approaches to inhibiting the STAT3 protein have been developed, and amongst its promising inhibitors is PPA and its derivatives [12,13,17,18,19,20,21].

PPA derivatives are novel little-studied compounds, and therefore the study of their effect on the signaling pathways of cell transduction, the activity of which is associated with the survival and resistance of tumor cells, is an important area. The key signaling pathways associated with proliferation, apoptosis, differentiation, and also underlying the emergence of drug resistance, include MAPK signaling [22], PI3K/Akt/mTOR signaling [23,24], and NF-κB signaling [25]. For a detailed assessment of the effect of PPA derivatives on signaling pathways, LuminexxMAP technology is used in this work [26,27].

This article includes information on the synthesis of novel PPA derivatives, a biological evaluation of novel and known derivatives, including the measurements of the cytotoxic effects, and the ability to overcome drug resistance and modulate signaling pathways.

## 2. Results and Discussion

### 2.1. Chemistry

**Scheme 1:**
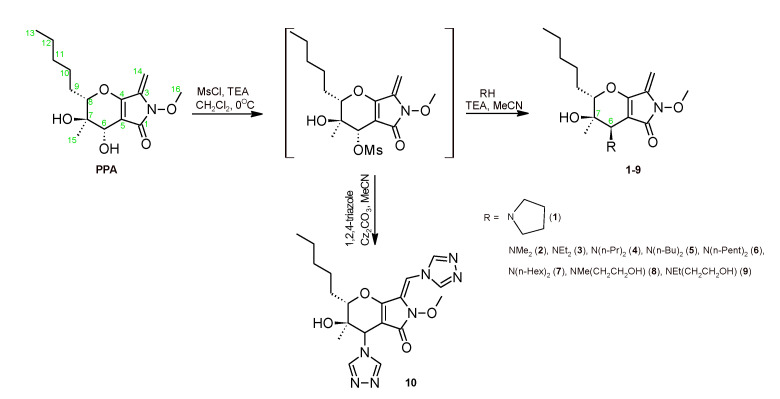
Synthesis of target compounds. The atom numbering scheme is that of Clardy et al. [14] and differs from the IUPAC one.

Scheme 1 shows the synthesis of target compounds **1**–**10**. PPA was mesylated with MsCl and TEA in CH_2_Cl_2_ and the mesylate formed was used in the next step without purification (Scheme 1) [19].

Secondary and cyclic amines were used to treat the mesylate. The reactions proceeded in the presence of TEA in acetonitrile at room temperature and led to amino derivatives **1–9** in a 16–43% yield. The ROESY spectrum of **5** showed a correlation between the methyl protons at δ 1.05 (H-7) and the proton at δ 3.14 (H-6), confirming the inversion of configuration of the C-6 atom (see Supplementary Materials). Products **1** and **10** were synthesized by our research group in 2017 and 2021 [19,21], respectively, and herein we show their new biological activities.

### 2.2. Biological Activities

#### 2.2.1. Assessment of the Cytotoxicity Level of Compounds on a Panel of Tumor Cell Cultures

##### Assessment of the Cytotoxicity Level on Cultures of Tumor Cells

At the first stage, the level of cytotoxicity of the obtained compounds was assessed on a panel of tumor cultures of various histogenesis (Table 1 and Table 2). The obtained compounds differ in the level of cytotoxicity, which, in most cases, is higher than the control substance etoposide. The synthesized compounds are especially active against cell cultures of tumors of the hematopoietic system, in particular, against multiple myeloma cells (NCI-H929) and acute T-cell leukemia (Jurkat). As the number of carbon atoms in the alkyl groups in the dialkylamine substituents increased, the activity of the substances decreased, so the IC50 was not checked for products **4**,**6**,**7**.

##### Assessment of the Cytotoxicity Level in Primary Cultures of Soft Tissue Sarcomas

Furthermore, the cytotoxicity level of the several most active compounds in the primary cultures of soft tissue sarcomas (SMTs) was assessed [28], i.e., cultures obtained from samples of SMT tumors, and passed several times outside the body. It is believed that such cultures have a molecular profile that corresponds more with cells from the human body, in comparison with that of stable cultures of tumor cells [29] (Table 3).

The synthesized compounds have cytotoxic activity against primary cultures of SMT of various histological forms.

##### Overcoming Drug Resistance of Tumor Cells

**Scheme 2:**
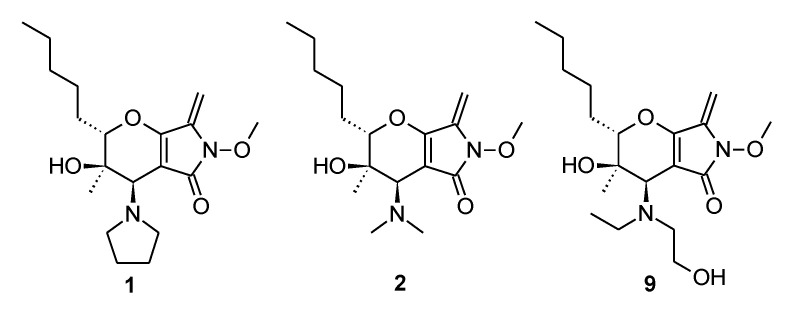
The structure of compounds **1**, **2**, and **9**.

**Figure 1:**
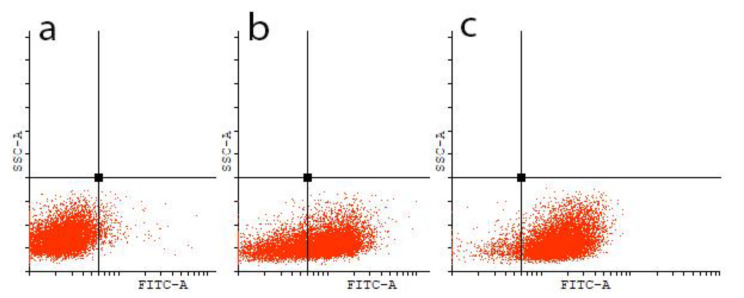
Expression of the P-gp protein in cells (**a**) K562, (**b**) K562/i-S9, and (**c**) K562/i-S9_Dox. Flow cytometry results from one of four runs. Cells are fixed and stained with a monoclonal antibody FITC Mouse Anti-Human P-glycoprotein, clone 17F9.

At the next stage, the cytotoxic activity of compounds **1**, **2**, and **9** (Scheme 2) was investigated on several pairs of cell lines that differ in the expression of P-gp and the method by which they were obtained. K562/i-S9 cells are the result of the *ABCB1* transfection into K562 cells, which increases P-gp expression, but should not seriously affect other signaling pathways that could lead to drug resistance. K562/i-S9_Dox cells were obtained from K562/i-S9 by selection with low concentrations of doxorubicin to increase P-gp expression. Subline HBL-100/Dox is a derivative of HBL-100 cells, obtained by long-term selection with increasing concentrations of doxorubicin, which led not only to an increase in P-gp expression, but also to a change in the signaling pathways in this subline. Cells of the K562/i-S9_Dox subline express the highest level of P-gp: 94.7% ± 2.3 (*p* < 0.0001 relative to K562, and *p* = 0.04 relative to K562/i-S9). In cells of the K562/i-S9 line, the expression of P-gp is 80% ± 8.2 (*p* < 0.0001 relative to K562). At 0.7% ± 0.5, P-gp is practically not expressed in K562 cells (Figure 1).

The described models were used to study the ability of the obtained compounds to overcome drug resistance associated with the overexpression of P-gp. As can be observed from Table 4, these cultures differ in resistance to dox, so K562/i-S9 and K562/i-S9_Dox cells are 11 (*p* = 0.0002) and 28 (*p* < 0.0001) times more resistant than K562, respectively. At the same time, K562/i-S9_Dox is 2.6 times more resistant than K562/i-S9 (*p* = 0.0002), which corresponds to a higher expression of P-gp in the K562/i-S9_Dox subline. The obtained compounds **1**, **2** and **9** have a cytotoxic effect on cells, with the same efficiency, regardless of the overexpression of P-gp. However, we noted a slightly greater sensitivity of K562/i-S9 cells to 1 (*p* = 0.015) relative to K562, and a tendency towards increased sensitivity to **9** (*p* = 0.08) relative to K562.

Similar results were obtained for the HBL-100 and HBL-100/Dox line pairs (Table 5). HBL-100/Dox cells were 556 times and 23 times more resistant to doxorubicin and etoposide, respectively (*p* < 0.0001), but there was no difference in resistance to **1** and **2** between cells. Only for **9** is there a tendency towards a slightly higher resistance of HBL-100/Dox cells compared to HBL-100.

Thus, the compounds studied in this work overcome drug resistance associated with P-gp overexpression, which has been shown in several pairs of sensitive and resistant tumor cell lines.

##### Interaction of the Obtained Compounds with the P-Glycoprotein

**Figure 2:**
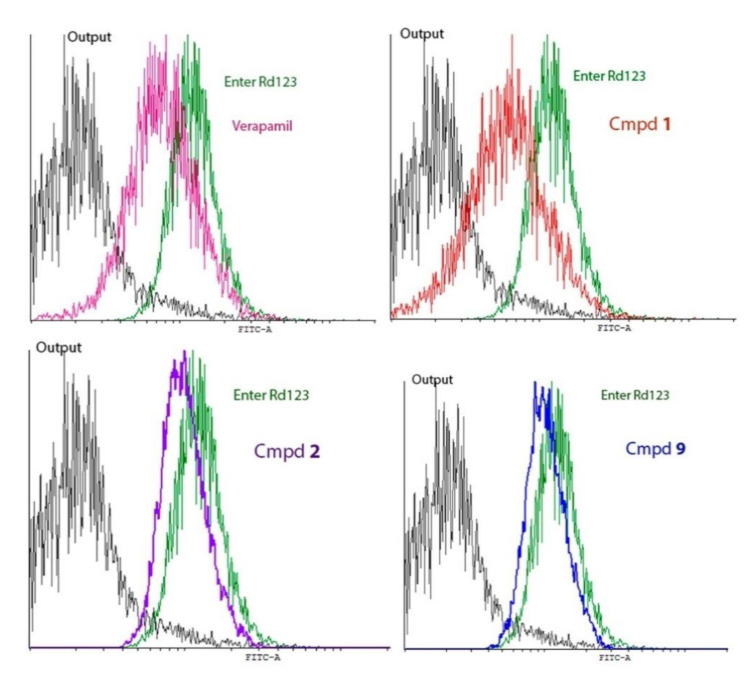
Functional test of the rhodamine 123 (Rh123) release from K562/i-S9_Dox cells via the activity of the P-glycoprotein. Histogram of cell luminosity intensity in the FITC-A channel. Green curve—luminosity of cells after the Rh123 entrance after 20 min of incubation; black curve—luminosity of cells after the release of Rh123 after 30 min of incubation in a pure medium; pink curve—luminosity of cells with the addition of verapamil; red curve—**1**; purple curve—**2**; and blue curve—**9** (incubation with preparations for 30 min).

We investigated the potential interaction of some synthesized compounds with P-gp (Table 6, Figure 2) in classical experiments on the release of rhodamine Rd123 (fluorescent agent, P-gp substrate). After 20 min of incubation with Rd123, almost 100% of the cells of the K562/i-S9_Dox line are stained, but after removing Rd123 from the mixture, after about 30 min, the number of luminous cells drops to 17%, because P-gp removes Rd123 from the cells. However, the addition of verapamil inhibits this process, and 90% of the cells remain luminous. All the investigated compounds act in the same direction, while substances **2** and **9** act with an efficiency similar to that of verapamil: the number of luminous cells is 82% and 80%, respectively. Compound **1** has a weaker interaction with P-gp than verapamil; the number of luminous cells is 73%.

The experiments show that the synthesized compounds modulate the activity of P-gp, significantly reducing it, with approximately the same efficiency as the classical inhibitor verapamil.

#### 2.2.2. Influence of Phaeosphaeride A Derivatives on the Key Signaling Pathways

LuminexxMAP technology was used to assess the effects of compounds **1**, **2**, **5**, **9**, **10** and PPA (the most active and the most inactive among all PPA derivatives) on the activation of intracellular kinase cascades in A431 cells. A431 cells are characterized by a high inducibility of transcription factors STAT3 and STAT5. In turn, phaeosphaeride A and its derivatives are probably inhibitors of the *STAT3* gene [14]. Therefore, the A431 cell line was chosen as a model for searching for possible targets for the action of phaeosphaeride A derivatives. Table 7 presents IC50 data for phaeosphaeride A derivatives on A431 cells.

We used the MILLIPLEX MAP Multi-Pathway Magnetic Bead 9-Plex-Cell Signaling Multiplex Assay, which allows the simultaneous detection of nine phosphorylated proteins that are markers of the main intracellular signaling pathways (phosphorylation site is indicated in parentheses), a universal transcription factor that controls the expression of immune response genes; apoptosis and cell cycle NFκB (pS536); cAMP-dependent transcription factor (CREB (pS133); mitogen-activated kinase p38 (pT180/pY182); stress-activated protein kinase JNK (pT183/pY185); ribosomal SK; transcription factors STAT3 (pS727) and STAT5A/B (pY694/699); protein kinase B (Akt) (pS473); and kinase regulated by extracellular signals ERK1/2 (pT185/pY187).

The effect of various concentrations of PPA derivatives on the cell culture was studied using xCelligence RTCA equipment (AceaBioscience, San Diego, CA, USA). The concentration closest to the IC50 (10 μM) was chosen as the experimental concentration. The selected concentration of phaeosphaeride A derivatives allows for the assessment of the early effect of compounds on cells and compares their effectiveness. In this experiment, the signaling pathways Akt (pSer473), p70S6K (pThr412), NFkB (p65-pSer536), and CREB (pSer133) were not activated (Table 8). The stress-activated signaling pathways ERK/MAP kinase 1/2 (pThr185/pTyr187), JNK (pThr183/pTyr185), and p38 (pThr180/pTyr182) were activated for these compounds.

**Figure 3:**
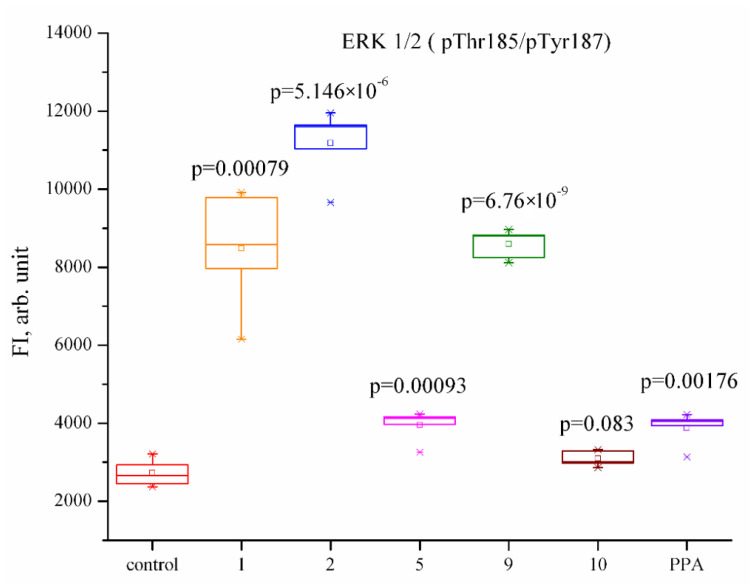
Box and Whisker Plot of the fluorescence intensity of the ERK 1/2 (pThr185/pTyr187) active form. A431 epidermoid carcinoma cells were treated with 10 µM compounds **1**, **2**, **5**, **9**, **10**, or PPA for 1 h. Cell lysates were analyzed by MILLIPLEX MAP Multi-Pathway Magnetic Bead 9-Plex kit (48–680 MAG, Merck) using a Bio-Rad 200 analyzer. Statistical analysis was performed using one-way ANOVA (Bio-Plex Data Pro™ Software). The differences were considered statistically significant at *p* < 0.05.

The fluorescence intensity of the active forms ERK1, JNK, and p38 increased by 1.5–3 times under the action of **1**, **2**, and **9**, i.e., substances with the greatest cytotoxic effect (Figure 3). Compound **2** exhibited the maximum degree of activation. These pathways were only slightly activated under the action of **5**, **10**, and PPA, which showed a significantly lower cytotoxic effect on tumor cultures.

All compounds, with the exception of **2**, did not significantly affect the activation of the transcription factors STAT5A/B (pTyr694/699) and STAT3 (pSer727). Compound **2** slightly activated these proteins, probably through the strong activation of the ERK1, JNK, and p38 proteins [30].

The set of activated kinase cascades suggests that oxidative stress is the main mechanism of the toxic action of PPA derivatives, leading to cell death [31,32,33]. In further work, it would be expedient to evaluate the markers of oxidative stress under the action of PPA derivatives and the kinetics of their activation on various cell lines.

## 3. Materials and Methods

### 3.1. Chemistry

The 1H NMR spectra were acquired on a Bruker AVANCE III 400 MHz NMR spectrometer in CDCl_3_. Optical rotations were acquired on an Optical Activity Polaar 3005 Polarimeter using a 2.5 cm cell with an Na 589 nm filter and the concentration of the samples was denoted as c. The mass spectra data were acquired on a Thermo Scientific TSQ Quantum Access Max Mass spectrometer. High-resolution mass spectra (HRMS) were acquired on a LTQ OrbitrapVelos spectrometer and on a Bruker MicrOTOF. FTIR spectra were acquired on a Shimadzu IR Affinity-1 spectrometer. Organic solvents used were dried by standard methods when necessary. Commercially available reagents were used without further purification. All reactions were monitored by TLC with EMD/Merck KGaA silica gel-coated plates, with visualization by UV light and by charring with 0.1% ninhydrin in EtOH. Column chromatography was performed using Merck 60 Å 70-230 mesh silica gel. The optical density was determined using a Multiskan FC spectrophotometer (Thermo Scientific, Waltham, MA, USA) at a wavelength of 540 nm when using the MTT assay.

General Procedure for the Synthesis of Compounds **2**–**9**
A mixture of the crude (2S,3S,4S)-3-hydroxy-6-methoxy-3-methyl-7-methylene-5-oxo-2-pentyl-2,3,4,5,6,7-hexahydropyrano[2,3-c]pyrrol-4-yl methane sulfonate (1 mmol), the corresponding secondary amine (1.5 mmol), and triethylamine (3 mmol) was stirred in dry acetonitrile (2 mL) at room temperature until the consumption of the starting material was complete as judged by TLC analysis (24 h). The reaction mixture was quenched with water and extracted with EtOAc (2 × 20 mL). The organic extract was washed with brine, dried over magnesium sulfate, and concentrated in vacuo. The crude product was purified by flash chromatography (DCM/methanol).
(2S,3R,4R)-4-(dimethylamino)-3-hydroxy-6-methoxy-3-methyl-7-methylene-2-pentyl-3,4,6,7-tetrahydropyrano[2,3-c]pyrrol-5(2H)-one (**2**). Yield 45%, yellow oil. −158.11 (c 1.11, CH_2_Cl_2_). 1H NMR (400 MHz, CDCl_3_) δ 5.26 (br s, 1H), 5.03 (d, J = 1.5 Hz, 1H), 4.99 (d, J = 1.5 Hz, 1H), 3.93 (s, 3H), 3.65–3.63 (m, 1H), 3.00 (s, 1H), 2.44 (br s, 6H), 2.00–1.91 (m, 1H), 1.69–1.53 (m, 2H), 1.44–1.31 (m, 5H), 1.04 (s, 3H), 0.91 (t, J = 6.7 Hz, 3H). 13C NMR (101 MHz, CDCl_3_) δ 167.18 (s), 158.35 (s), 137.11 (s), 101.74 (s), 91.58 (s), 83.24 (s), 68.47 (s), 64.58 (s), 62.74 (s), 31.86 (s), 28.42 (s), 26.59 (s), 22.69 (s), 20.18 (s), 14.19 (s). IR (KBr) 3430, 2955, 2930, 2859, 1723, 1635, 1436, 1370, 1265, 1149, 986, 775 cm^−1^. HRMS [M + H]+calcd for C_17_H_29_N_2_O_4_ 325.21218, found 325.21206.
(2S,3R,4R)-4-(diethylamino)-3-hydroxy-6-methoxy-3-methyl-7-methylene-2-pentyl-3,4,6,7-tetrahydropyrano[2,3-c]pyrrol-5(2H)-one (**3**). Yield 50%, yellow oil. −182.36 (c 1.15, CH_2_Cl_2_). 1H NMR (400 MHz, CDCl_3_) δ 5.23 (s, 1H), 5.03 (d, J = 1.5 Hz, 1H), 4.98 (d, J = 1.5 Hz, 1H), 3.93 (s, 3H), 3.56–3.54 (m, 1H), 3.15 (s, 1H), 3.06–2.27 (m, 4H), 2.02–1.92 (m, 1H), 1.72–1.54 (m, 2H), 1.49–1.30 (m, 5H), 1.09 (br s, 6H), 1.05 (s, 3H), 0.92 (t, J = 6.8 Hz, 3H). 13C NMR (101 MHz, CDCl_3_) δ 167.05 (s), 158.53 (s), 137.16 (s), 102.33 (s), 91.43 (s), 83.26 (s), 67.93 (s), 64.52 (s), 60.14 (s), 48.97 (s), 45.76 (s), 31.85 (s), 28.49 (s), 26.62 (s), 22.69 (s), 19.98 (s), 14.20 (s). IR (KBr) 3207, 2961, 2932, 2860, 1723, 1635, 1468, 1379, 1268, 1149, 978, 764 cm^−1^. HRMS [M + H]+calcd for C_19_H_33_N_2_O_4_ 353.24348, found 353.24324.
(2S,3R,4R)-4-(dipropylamino)-3-hydroxy-6-methoxy-3-methyl-7-methylene-2-pentyl-3,4,6,7-tetrahydropyrano[2,3-c]pyrrol-5(2H)-one (**4**). Yield 40%, yellow oil. −205.07 (c 2.01, CH_2_Cl_2_). 1H NMR (400 MHz, CDCl_3_) δ 5.27 (s, 1H), 5.03 (d, J = 1.4 Hz, 1H), 4.99 (d, J = 1.4 Hz, 1H), 3.93 (s, 3H), 3.58 (m, 1H), 3.14 (s, 1H), 2.98 (br s, 1H), 2.46 (m, 3H), 1.98–1.90 (m, 1H), 1.64–1.34 (m, 11H), 1.04 (s, 3H), 0.92–0.89 (m, 9H). 13C NMR (101 MHz, CDCl_3_) δ 167.08 (s), 158.51 (s), 137.19 (s), 102.36 (s), 91.48 (s), 83.27 (s), 83.17 (s), 68.20 (s), 64.53 (s), 61.00 (s), 58.02 (s), 54.07 (s), 31.76 (s), 28.40 (s), 26.45 (s), 22.67 (s), 22.28 (s), 21.88 (s), 20.16 (s), 14.19 (s), 11.90 (s). IR (KBr) 3464, 2960, 2932, 2873, 1724, 1635, 1436, 1378, 1149, 1072, 914 cm^−1^. HRMS [M + H]+calcd for C_21_H_37_N_2_O_4_ 381.27478, found 381.27435.
(2S,3R,4R)-4-(dibutylamino)-3-hydroxy-6-methoxy-3-methyl-7-methylene-2-pentyl-3,4,6,7-tetrahydropyrano[2,3-c]pyrrol-5(2H)-one (**5**). Yield 39%, yellow oil. −178.71 (c 1.8, CH_2_Cl_2_). 1H NMR (400 MHz, CDCl_3_) δ 5.28 (s, 1H), 5.03 (d, J = 1.5 Hz, 1H), 4.99 (d, J = 1.5 Hz, 1H), 3.93 (s, 3H), 3.59–3.57 (m, 1H), 3.14 (s, 1H), 2.99 (br s, 1H), 2.70–2.26 (m, 3H), 1.99–1.91 (m, 1H), 1.67–1.31 (m, 15H), 1.05 (s, 3H), 1.05–0.90 (m, 9H). 13C NMR (101 MHz, CDCl_3_) δ 167.12 (s), 158.53 (s), 137.20 (s), 102.37 (s), 91.59 (s), 91.47 (s), 83.30 (s), 83.20 (s), 68.15 (s), 64.61 (s), 64.54 (s), 60.99 (s), 60.94 (s), 31.76 (s), 28.41 (s), 26.57 (s), 22.68 (s), 20.63 (s), 20.18 (s), 14.24 (s). IR (KBr) 3437, 2958, 2931, 2860, 1724, 1634, 1435, 1378, 1150, 1076, 1033, 914 cm^−1^. HRMS [M + H]+calcd for C_23_H_41_N_2_O_4_ 409.30608, found 409.30580.
(2S,3R,4R)-4-(dipentylamino)-3-hydroxy-6-methoxy-3-methyl-7-methylene-2-pentyl-3,4,6,7-tetrahydropyrano[2,3-c]pyrrol-5(2H)-one (**6**). Yield 40%, yellow oil. −179.86 (c 1.45, CH_2_Cl_2_). 1H NMR (400 MHz, CDCl_3_) δ 5.29 (s, 1H), 5.03 (d, J = 1.4 Hz, 1H), 4.99 (d, J = 1.4 Hz, 1H), 3.93 (s, 3H), 3.59–3.57 (m, 1H), 3.14 (s, 1H), 2.99 (br s, 1H), 2.70–2.22 (m, 3H), 2.00–1.91 (m, 1H), 1.62–1.25 (m, 19H), 1.05 (s, 3H), 0.93–0.89 (m, 9H). 13C NMR (101 MHz, CDCl_3_) δ 167.10 (s), 158.51 (s), 137.19 (s), 102.37 (s), 91.46 (s), 83.27 (s), 83.16 (s), 68.15 (s), 64.53 (s), 60.98 (s), 60.92 (s), 55.98 (s), 52.11 (s), 31.73 (s), 29.62 (s), 29.00 (s), 28.41 (s), 26.54 (s), 22.76 (s), 22.68 (s), 20.18 (s), 14.19 (s). IR (KBr) 3485, 2957, 2930, 2860, 2072, 1724, 1634, 1436, 1378, 1150, 1079, 915 cm^−1^. HRMS [M + H]+calcd for C_25_H_45_N_2_O_4_ 437.33738, found 437.33701.
(2S,3R,4R)-4-(dihexylamino)-3-hydroxy-6-methoxy-3-methyl-7-methylene-2-pentyl-3,4,6,7-tetrahydropyrano[2,3-c]pyrrol-5(2H)-one (**7**). Yield 41%, yellow oil. −185.98 (c 0.97, CH_2_Cl_2_). 1H NMR (400 MHz, CDCl_3_) δ 5.28 (s, 1H), 5.03 (d, J = 1.4 Hz, 1H), 4.98 (d, J = 1.4 Hz, 1H), 3.93 (s, 3H), 3.60–3.57 (m, 1H), 3.13 (s, 1H), 2.98 (br s, 1H), 2.67–2.29 (m, 3H), 1.99–1.91 (m, 1H), 1.65–1.29 (m, 23H), 1.04 (s, 3H), 0.93–0.86 (m, 9H). 13C NMR (101 MHz, CDCl_3_) δ 167.11 (s), 158.51 (s), 137.20 (s), 102.39 (s), 91.44 (s), 83.30 (s), 83.20(s), 68.15 (s), 64.61 (s), 64.53 (s), 60.99 (s), 60.94 (s), 56.15 (s), 52.34 (s), 31.91 (s), 31.75 (s), 29.28 (s), 28.80 (s), 28.42 (s), 27.12 (s), 26.57 (s), 22.77 (s), 22.69 (s), 20.18 (s), 14.18 (s). IR (KBr) 3474, 2957, 2929, 2858, 2075, 1724, 1634, 1435, 1378, 1151, 1081, 914 cm^−1^. HRMS [M + H]+calcd for C_27_H_49_N_2_O_4_ 465.36868, found 465.36853.
(2S,3R,4R)-3-hydroxy-4-[(2-hydroxyethyl)(methyl)amino]-6-methoxy-3-methyl-7-methylene-2-pentyl-3,4,6,7-tetrahydropyrano[2,3-c]pyrrol-5(2H)-one (**8**). Yield 63%, yellow oil. −151.76 (c 2.1, CH_2_Cl_2_). 1H NMR (400 MHz, CDCl_3_) δ 5.10 (d, J = 1.7 Hz, 1H), 5.05 (d, J = 1.6 Hz, 1H), 4.82 (br s, 1H), 4.01–3.95 (m, 4H), 3.65–3.62 (m, 2H), 3.50 (s, 1H), 3.09–2.90 (m, 2H), 2.42 (s, 3H), 2.02–1.93 (m, 1H), 1.69–1.57 (m, 2H), 1.44–1.33 (m, 5H), 1.06 (s, 3H), 0.92 (t, J = 6.8 Hz, 3H). 13C NMR (101 MHz, CDCl_3_) δ 167.72 (s), 158.61 (s), 136.70 (s), 101.63 (s), 92.74 (s), 83.18 (s), 69.57 (s), 64.76 (s), 61.20 (s), 59.22 (s), 39.75 (s), 32.06 (s), 31.83 (s), 29.84 (s), 28.23 (s), 26.54 (s), 22.68 (s), 19.75 (s), 14.17 (s). IR (KBr) 3420, 2956, 2929, 2859, 1719, 1635, 1438, 1378, 1193, 1081, 1021, 951, 913 cm^−1^. HRMS [M + H]+calcd for C_19_H_30_N_2_O_5_ 355.22275, found 355.22267.
(2S,3R,4R)-4-[ethyl(2-hydroxyethyl)amino]-3-hydroxy-6-methoxy-3-methyl-7-methylene-2-pentyl-3,4,6,7-tetrahydropyrano[2,3-c]pyrrol-5(2H)-one (**9**). Yield 45%, yellow oil. −197.38 (c 1.91, CH_2_Cl_2_). 1H NMR (400 MHz, CDCl_3_) δ 5.10 (s, 1H), 5.05 (s, 1H), 4.71 (br s, 1H), 3.94 (br s, 4H), 3.62–3.59 (m, 1H), 3.19–2.52 (m, 4H), 2.02–1.90 (m, 1H), 1.69–1.55 (m, 4H), 1.45–1.32 (m, 5H), 1.07 (br s, 6H), 0.91 (t, J = 6.8 Hz, 3H). 13C NMR (101 MHz, CDCl_3_) δ 167.70 (s), 158.60 (s), 136.76 (s), 102.19 (s), 92.59 (s), 83.07 (s), 69.10 (s), 64.70 (s), 60.78 (s), 59.36 (s), 55.50 (s), 50.96 (s), 31.81 (s), 28.25 (s), 26.56 (s), 22.67 (s), 19.72 (s), 14.39 (s), 14.19 (s). IR (KBr) 3430, 2958, 2931, 2860, 1720, 1634, 1438, 1438, 1379, 1336, 1151, 1054, 990, 913, 743 cm^−1^. HRMS [M + H]+calcd for C_19_H_33_N_2_O_5_ 369.23840, found 369.23828.

### 3.2. Biological Assay Methods

#### 3.2.1. Assessment of the Level of Compounds Cytotoxicity in a Panel of Tumor Cell Cultures

##### Tumor Cell Cultures

Cell cultures used in the work: adhesive cultures HBL-100 cells (cells of a normal mammary gland epithelium, immortalized by the SV40 virus and acquired tumorigenicity [34]); MCF-7 breast cancer cells; PC-3 prostate adenocarcinoma cells; HCT-116 colorectal cancer cells; non-small cell lung cancer cells A549; human embryonic kidney cells HEK293; suspension cultures (NCI-H929 and RPMI8226 multiple myeloma cells), K562 chronic myeloid leukemia cells; THP-1 acute monocytic leukemia cells; and Jurkat acute T-cell leukemia cells. We also used several primary cultures of soft tissue sarcomas at 3–4passeges, kindly provided by T.I. Fetisov (The Ethics Committee of N.N. Blokhin NMRCO, protocol code 18-28-09095 and date of approval 28 September 2018) [35].

Modifications of cell lines: K562/i-S9 is a clone obtained after transfection of K562 cells with the ABCB1 (MDR1) gene-encoding P-glycoprotein [36]. Cells HBL-100/Dox and K562/i-S9_Dox are cells obtained by long-term selection with increasing concentrations of doxorubicin (Sigma-Aldrich, St. Louis, MO, USA) from HBL-100 and K562/i-S9 cells, respectively. MCF-7, A594 cells, and primary cultures of soft tissue sarcomas were cultured in DMEM medium (PanEko, Russia), PC-3 and HCT-116 cells and all suspension cultures in RPMI1640 medium (PanEko, Russia) supplemented with 10% fetal bovine serum (GE Healthcare LifeSciences, USA), and gentamicin at a concentration of 40 μg/mL at 37 °C and 5% CO_2_.

##### MTT Test

Cells MCF-7, PC-3, A549, and HCT-116, and primary cultures of STSwere seeded in 96-well plates, 6 × 10^3^ cells per well in 90 μL of culture medium. Suspension cultures were seeded at a concentration of 25 × 10^3^ cells per well in 135 μL of medium. The test substances were dissolved in DMSO (Applichem, Council Bluffs, IA, USA) until a concentration of 1 × 10^−^^2^ M was obtained; for the subsequent dilution of the substances, a serum-free culture medium was used. The next day, for adhesive cultures, and on the same day, for suspension cultures, substances were added to the plates in a volume of 10 μL (adhesive cultures) and 15 μL (suspension cultures) per well at various concentrations; each concentration was added in 3–4 repetitions. The cells were cultured in the presence of substances for 48 h. Then, MTT reagent (PanEko, Moscow, Russia) was added to the wells at a concentration of 5 mg/mL in a volume of 20 μL per well. After 1.5–2 h, the medium with the reagent was removed, and the precipitate of the crystals of formazan was dissolved in 60 μL of DMSO. The optical density was determined using a Multiskan FC spectrophotometer (Thermo Scientific, Waltham, MA, USA) at a wavelength of 540 nm.

Based on the results of the experiments, a schedule was plotted of the dependence of the percentage of surviving cells (from control) on the concentration of the compound, and the sought concentration IC50 was calculated. The experiments were carried out in 3–4 times.

##### Flow Cytometry

To assess the expression of P-gp in K562, K562/i-S9, and K562/i-S9_Dox cells, 5 × 10^5^ cells were fixed in 4% formaldehyde solution, then washed twice in PBS, after 30 min they were incubated with monoclonal antibody FITC Mouse Anti-Human P-glycoprotein, clone 17F9 (BD Biosciences, San Jose, CA, USA) was washed twice to remove antibodies in PBS. The number of luminous cells was analyzed using a Becton Dickinson FACScan flow cytometer. The results were analyzed using Diva and CyFlodgic software.

##### Evaluation of the Release of Rhodamine 123 (Rd123) from Cells with an Overexpression of P-Glycoprotein

To assess the interaction of the investigated compounds with P-gp, we used the test for the release of rhodamine 123 (Rd123) (Sigma-Aldrich, St. Louis, MO, USA) from cells with P-gp overexpression K562/i-S9_Dox. The principle of the test is as follows: Rd123 is a fluorophore and a known P-gp substrate. During incubation, Rd123 enters the cells by simple diffusion, and then Rd123 begins to interact with P-gp, which releases it from the cell. Cells in which Rd123 is present are stained and can be detected by flow cytometry. The more efficiently P-gp works, the less stained cells in the population remain over time. If inhibitors or modulators of P-gp activity are added to the cells, then the cells remain stained; if substances do not interact with P-gp, then the cell population loses its luminosity.

The experiment used K562/i-S9_Dox cells with P-gp overexpression. Cells (5 × 10^5^ per point) were incubated with Rd123 at a concentration of 3.5 μg/mL for 20 min in serum-free RPMI1640 medium. Furthermore, the efficiency of Rd123 entry into cells was assessed. Subsequently, the cells were washed once from Rd123 in a PBS solution, and the precipitate was divided into several parts; the cells were placed in a clean serum-free medium to assess the efficiency of Rd123 release, as well as in a serum-free medium with the addition of the following compounds at a concentration of 20 μM: verapamil (a classic inhibitor of P-gp) and test compounds **1**, **2**, and **9**. Incubation with substances took place for 35 min in 1 mL of serum-free medium. Then, the cells were precipitated, washed with PBS solution, and the % of stained cells in the population were assessed on a flow cytometer.

##### Statistical Analysis

The results were presented as the mean ± SD. GraphPad Prism 6.0 was used for statistical analysis. The statistical significance was evaluated by unpaired *t*-test.

#### 3.2.2. Evaluation of the Effect of Phaeosphaeride A Derivatives on Key Signaling Pathways

To determine the integral cellular response using the xCelligence RTCA equipment (AceaBioscience, Santa Clara, CA, USA), 20 thousand cells were inoculated into a well of a specialized plate allowing the determination of the cell index in real time and cultured in complete growth medium. The next day after the seeding, the medium was replaced with an experimental one (compounds at concentrations of 50, 20, 10, 5, 2, 1, and 0.5 μm). The cell index was monitored for 3 days after the addition of the xenobiotic. The concentration closest to the IC50 (10 μM) was chosen as the experimental concentration.

We used a MILLIPLEX MAP Multi-Pathway Magnetic Bead 9-Plex-Cell Signaling Multiplex Assay kit (Merck/Millipore), according to the manufacturer’s protocol. A431 cells were cultured in DMEM with 10% FBS in 24-well plates. Compounds were added to the subconfluent cells at a concentration of 10 μM in DMEM medium for 1 h. Each compound was added in 5 experimental repeats. Cell lysates were prepared with lysis buffer (Millipore) with protease inhibitors (Complete, Roche); the protein concentration in the lysates was determined by the Lowry method. In samples equalized in protein concentration, the active forms of kinase cascades were determined using a Bio-Plex 200 fluorescent analyzer (Bio-Rad).

## 4. Conclusions

Thus, the obtained PPA derivatives can overcome the drug resistance of tumor cells associated with the overexpression of P-glycoprotein by modulating the work of this transporter, which makes them promising in terms of creating new anticancer drugs aimed at treating resistant malignant neoplasms. A potential mechanism of the toxic effect of PPA derivatives associated with oxidative stress has also been identified. In addition, the effective two-stage synthesis of the presented compounds based on natural substances makes the development of this project even more attractive.

## Figures and Tables

**Table 1:** IC50 value (μM) of compounds for adhesive tumor cultures of epithelial origin.

	HCT-116	MCF-7	PC3	A549	HEK293
Etoposide	21.0 ± 9.9	8.9 ± 2.3	27.1 ± 3.2	65.3 ± 5.2	2.0 ± 0.5
**1**	7.6 ± 0.9	3.9 ± 0.8	7.6 ± 0.9	14.0 ± 0.1	5.1 ± 0.7
**2**	4.2 ± 0.2	3.0 ± 0.8	5.9 ± 3.0	9.6 ± 2.1	4.2 ± 0.3
**5**	12.0 ± 0.9	7.4 ± 0.5	9.4 ± 1.0	23.4 ± 2.3	8.2 ± 0.9
**8**	7.5 ± 1.0	1.8 ± 0.4	3.3 ± 0.6	16.3 ± 0.9	3.8 ± 0.2
**9**	3.9 ± 0.1	2.9 ± 1.0	7.8 ± 0.1	8.9 ± 0.2	3.8 ± 0.4
**10**	not active	49.0 ± 5.3	not active	not active	41.0 ± 2.1

**Table 2:** IC50 value (μM) of compounds for suspension cultures derived from hematopoietic tumors.

	NCI-H929	THP-1	K562	RPMI8226	Jurkat
Etoposide	1.4 ± 0.5	1.8 ± 0.2	5.5 ± 3.0	7.2 ± 1.9	0.9 ± 0.3
**1**	0.7 ± 0.2	1.5 ± 0.4	3.8 ± 0.6	1.6 ± 0.3	1.5 ± 0.4
**2**	0.5 ± 0.1	0.9 ± 0.3	2.4 ± 0.7	1.6 ± 0.5	1.1 ± 0.1
**5**	2.9 ± 0.3	8.2 ± 0.6	6.5 ± 0.5	6.3 ± 0.3	3.7 ± 0.2
**8**	0.9 ± 0.3	1.8 ± 0.2	3.4 ± 0.3	1.5 ± 0.4	1.0 ± 0.3
**9**	0.6 ± 0.2	1.7 ± 0.2	3.7 ± 0.5	1.8 ± 0.2	1.3 ± 0.4
**10**	5.6 ± 0.7	42.0 ± 5.2	45.0 ± 5.4	51.0 ± 0.2	94.0 ± 22.6

**Table 3:** IC50 value (μM) of compounds for primary cultures of soft tissue sarcomas.

	CMT № 1	CMT № 2	CMT № 3
Histological Subtype	Extraskeletal Ewing’s Sarcoma	Malignant Schwannoma	Epithelial Sarcoma (Metastasis)
**1**	2.6 ± 1.1	2.5 ± 0.9	4.8 ± 0.9
**2**	1.6 ± 0.7	1.9 ± 0.5	7.8 ± 0.6
**8**	3.9 ± 0.8	2.3 ± 0.8	4.8 ± 0.5
**9**	2.9 ± 1.1	4.1 ± 1.1	13 ± 0.9

**Table 4:** IC50 value of compounds on sensitive K562 cells and resistant versions K562/i-S9 and K562/i-S9_Dox.

Compound	K562	K562/i-S9	K562/i-S9_Dox
Doxorubicin, µM	0.36 ± 0.17	3.9 ± 1.6	10.3 ± 1.2
**1**, µM	3.8 ± 0.6	2.4 ± 0.6	3.1 ± 0.8
**2**, µM	2.4 ± 0.7	1.6 ± 0.5	2.4 ± 0.9
**9**, µM	3.7 ± 0.5	2.8 ± 0.6	3.3 ± 0.6

**Table 5:** IC50 value of compounds for sensitive HBL-100 cells and resistant version HBL-100/Dox.

Compound	HBL-100	HBL-100/Dox	*p* Value
Doxorubicin, µM	0.23 ± 0.03	128.0 ± 7.4	*p* < 0.0001
Etoposide, µM	4.7 ± 0.9	111.3 ± 8.3	*p* < 0.0001
**1**, µM	6.2 ± 0.8	7.6 ± 0.6	0.26
**2**, µM	4.9 ± 1.0	6.6 ± 1.2	0.31
**9**, µM	6.5 ± 0.8	8.4 ± 0.4	0.06

**Table 6:** Release of Rd123 from K562/i-S9_Dox cells under the influence of the investigated compounds.

Sample	% Luminous Cells	Compounds vs. Release of Rh123	Compounds vs. Verapamil’s Rh 123 (Release)
Rh 123 entry	99.8 ± 0.3		
Rh123 release	17.1 ± 6.6		
Rh 123 (release) + verapamil	89.7 ± 8.1	*p* < 0.0001	
Rh123 (release) + **1**	73.4 ± 6.7	*p* < 0.0001	0.025
Rh 123 (release) + **2**	82.4 ± 3.0	*p* < 0.0001	0.13
Rh 123 (release) + **9**	80.2 ± 6.2	*p* < 0.0001	0.08

**Table 7:** IC50 value of compounds for A431 cells.

Compounds	1	2	5	9	10	PPA
IC50, µM	3.7	1.9	>50	4.6	>50	>50

**Table 8:** Influence of phaeosphaeride A derivatives on the level of protein active forms of key kinase cascades (the phosphorylation site is marked) in lysates of A431 cells, fluorescence intensity, rel. units (M ± mx).

Cmpd	Analyte (Fluorescence Intensity, Rel. Units (M ± mx))								
	Akt (pSer473)	CREB (pSer133)	ERK ½ (pThr185/pTyr187)	JNK (pThr183/pTyr185)	NF-kB (pSer536)	p38 (pThr180/pTyr182)	p70S6K (pThr389/412)	STAT3 (pSer727)	STAT5 (pTyr694/699)
Control	76.4 ± 16.9	154.5 ± 34.2	839.7 ± 248.9	50.9 ± 10.5	15.0 ± 1.5	567.7 ± 266.6	39.9 ± 7.2	70.7 ± 18.3	44.2 ± 10.2
**1**	112.6 ± 12.0	329.9 ± 48.4 *↑	4650.1 ± 509.0 *↑	239.9 ± 23.9 *↑	18.6 ± 1.5	2349.9 ± 755.5	51.7 ± 4.8	163.0 ± 20.7 *↑	115.4 ± 28.3
**2**	132.4 ± 17.9	610.0 ± 113.0 *↑	5074.6 ± 770.1 *↑	559.2 ± 31.5 *↑	18.2 ± 2.1	2795.4 ± 572.0 *↑	56.9 ± 9.9	272.3 ± 50.5 *↑	179.2 ± 12.6 *↑
**5**	100.9 ± 11.7	202.1 ± 26.3	1880.4 ± 81.7 *↑	83.1 ± 8.3 *↑	17.1 ± 0.8	1245.9 ± 342.8	70.6 ± 9.2 *↑	123.4 ± 8.3 *↑	75.7 ± 2.7 *↑
**9**	88.8 ± 10.7	339.8 ± 53.3 *↑	3276.9 ± 700.0 *↑	117.9 ± 15.1 *↑	13.9 ± 1.2	657.1 ± 287.5	38.8 ± 7.4	151.5 ± 28.7	69.6 ± 15.6
**10**	142.1 ± 9.5 *↑	584.8 ± 105.5 *↑	1864.9 ± 149.5 *↑	157.2 ± 15.4 *↑	15.4 ± 0.5	1783.6 ± 181.9 *↑	53.4 ± 4.0	178.9 ± 13.4 *↑	115.2 ± 16.9 *↑
PPA	123.5 ± 4.9 *↑	291.0 ± 32.8 *↑	995.4 ± 46.5	132.8 ± 9.1 *↑	19.3 ± 3.0	1459.1 ± 117.6 *↑	46.1 ± 2.9	128.8 ± 2.4 *↑	82.5 ± 3.5 *↑

Note: *—differences in the group are significant (*p* < 0.05) in comparison to the data of baseline studies (ANOVA); ↑—the indicator significantly increases.

## Data Availability

Data is contained within the article and Supplementary Material.

## Supplementary Materials

The following supporting information can be downloaded at: https://www.mdpi.com/article/10.3390/ph15040395/s1, Copies of 1H, 13C, ROESY NMR spectra, HRMS for compounds **2**–**9** (Figures S1–S25), markers of key signaling pathways for compounds **1**, **2**, **5**, **9**, **10**, **PPA** (Figures S26–S34).
