# Supplementary material for: Natural Phaeosphaeride A Derivatives Overcome Drug Resistance of Tumor Cells and Modulate Signaling Pathways
Source: Pharmaceuticals (Basel). 2022 Mar 24;15(4):395. doi: 10.3390/ph15040395 (PMC9030166; doi:10.3390/ph15040395)
Supplement: Supplementary file 1 [file pharmaceuticals-15-00395-s001.zip › pharmaceuticals-1627850-supplementary.pdf]

Natural Phaeosphaeride A Derivatives Overcome Drug Resistance of Tumor Cells and Modulate Signaling Pathways

Victoria Abzianidze [1,*], Natalia Moiseeva [2], Diana Suponina [1], Sofya Zakharenkova [1], Nadezhda Rogovskaya [1], Lidia Laletina [2], Alvin Holder [3,*], Denis Krivorotov [1], Alexander Bogachenkov [1], Alexander Garabadzhiu [4], Anton Ukolov [1] and Vyacheslav Kosorukov [5]

[1] Research Institute of Hygiene, Occupational Pathology and Human Ecology, Federal Medical Biological Agency, p/o Kuz'molovsky, 188663 Saint Petersburg, Russia; dina.lykina.97@mail.ru (D.S.); sofya.zakharenkova@gmail.com (S.Z.); nadin-r@mail.ru (N.R.); denhome@bk.ru (D.K.); alexterve@gmail.com (A.B.); antonukolov@gmail.com (A.U.)
[2] Laboratory of Tumor Cell Genetics, Institute of Carcinogenesis, N.N. Blokhin National Medical Research Center of Oncology of the Ministry of Health of the Russian Federation, 115478 Moscow, Russia;
n.i.moiseeva@gmail.com (N.M.); panlidia@gmail.com (L.L.)
[3] Department of Chemistry and Biochemistry, Old Dominion University, 4541 Hampton Boulevard,
Norfolk, VA 23529, USA
[4] Saint Petersburg State Technological Institute (Technical University), 190013 Saint Petersburg, Russia;
gar-54@mail.ru
[5] Laboratory of Transgenic Drugs, N.N. Blokhin National Medical Research Center of Oncology of the
Ministry of Health of the Russian Federation, 115478 Moscow, Russia; atgtga@mail.ru
* Correspondence: vvaavv@mail.ru (V.A.); aholder@odu.edu (A.H.); Tel.: +7-981-249-0902 (V.A.);
+1-757-683-7102 (A.H.)

Spectra

(2*S*,3*R*,4*R*)-4-(dimethylamino)-3-hydroxy-6-methoxy-3-methyl-7-methylene-2-pentyl-3,4,6,7-tetrahydropyrano[2,3-*c*]pyrrol-5(2*H*)-one (**2**)

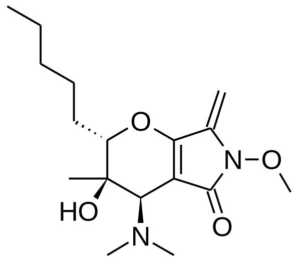

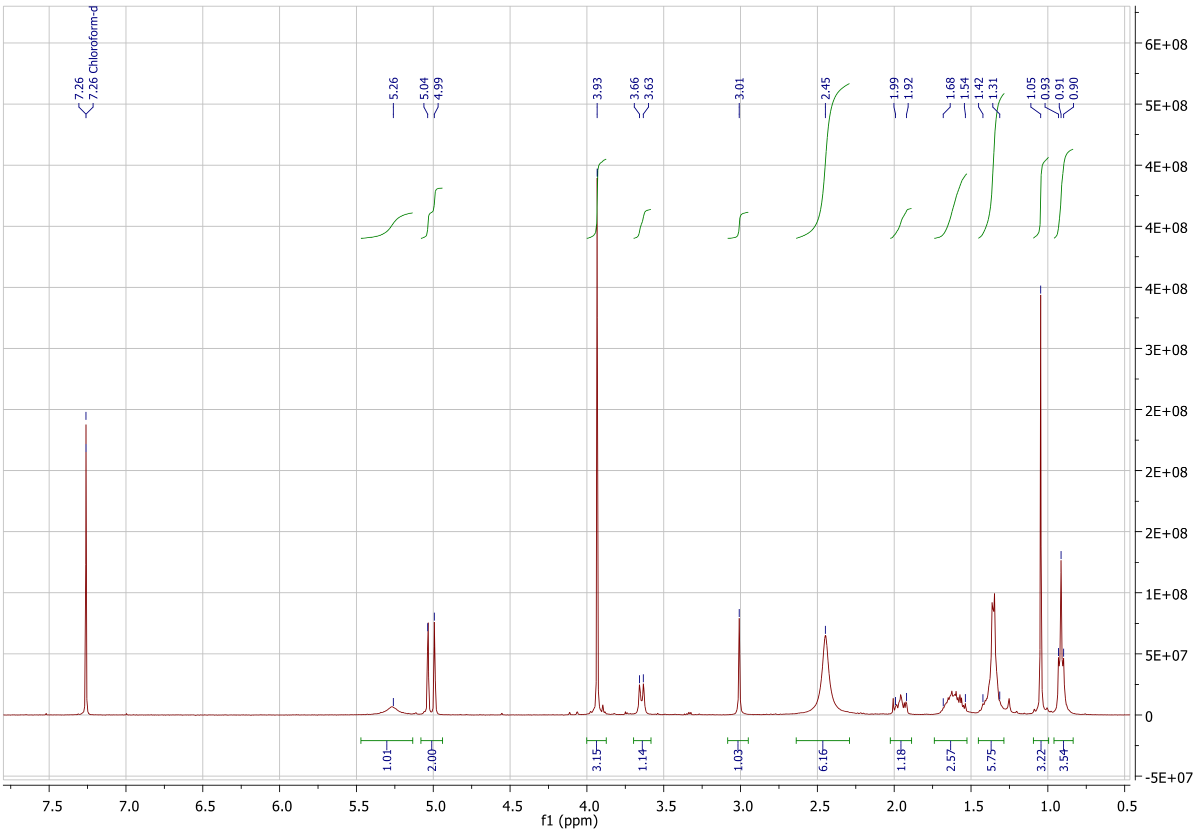

Figure S1. ^{1}H-NMR of **2**.

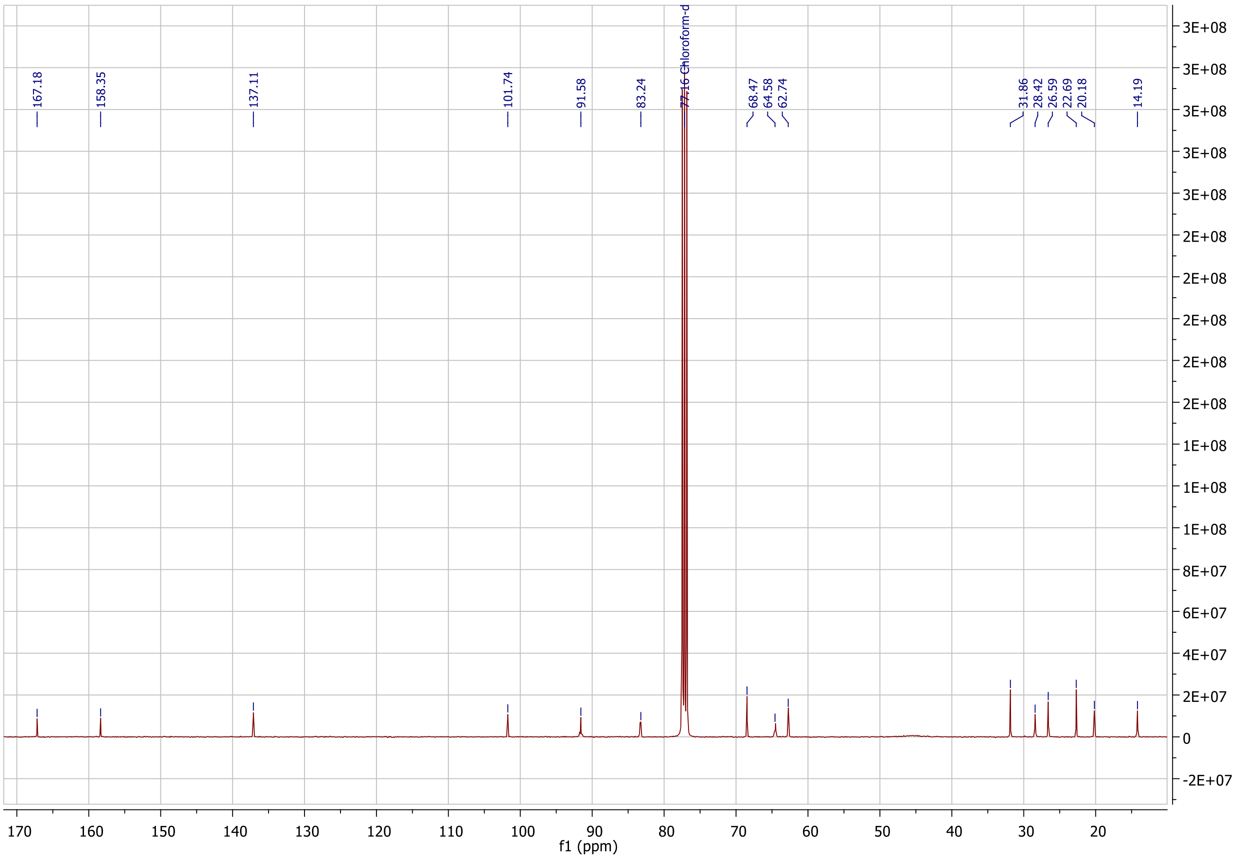

Figure S2. ^{13}C-NMR of **2**.

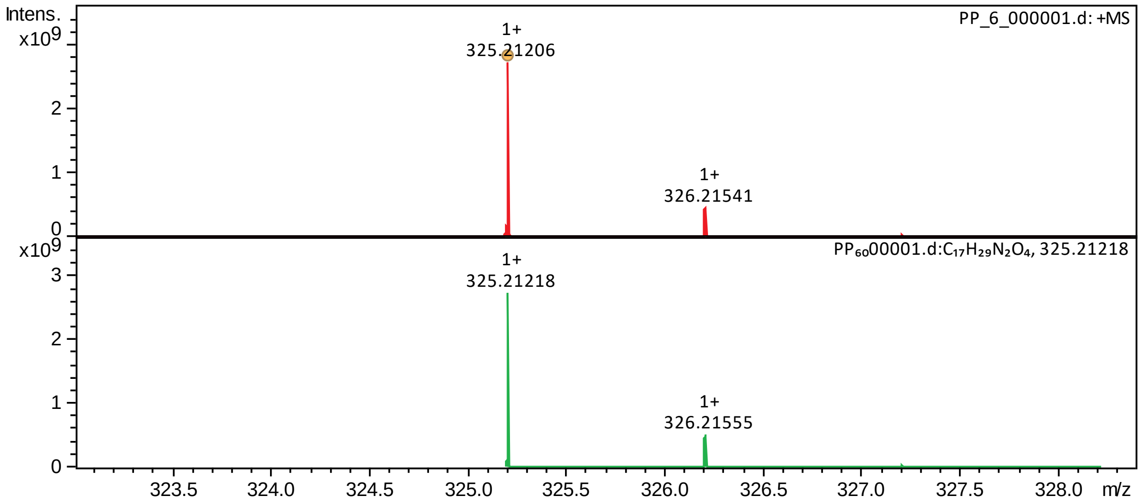

Figure S3. HRMS of **2**.

(2*S*,3*R*,4*R*)-4-(diethylamino)-3-hydroxy-6-methoxy-3-methyl-7-methylene-2-pentyl-3,4,6,7-tetrahydropyrano[2,3-*c*]pyrrol-5(2*H*)-one (**3**)

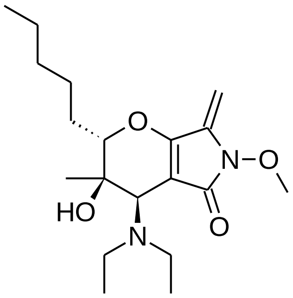

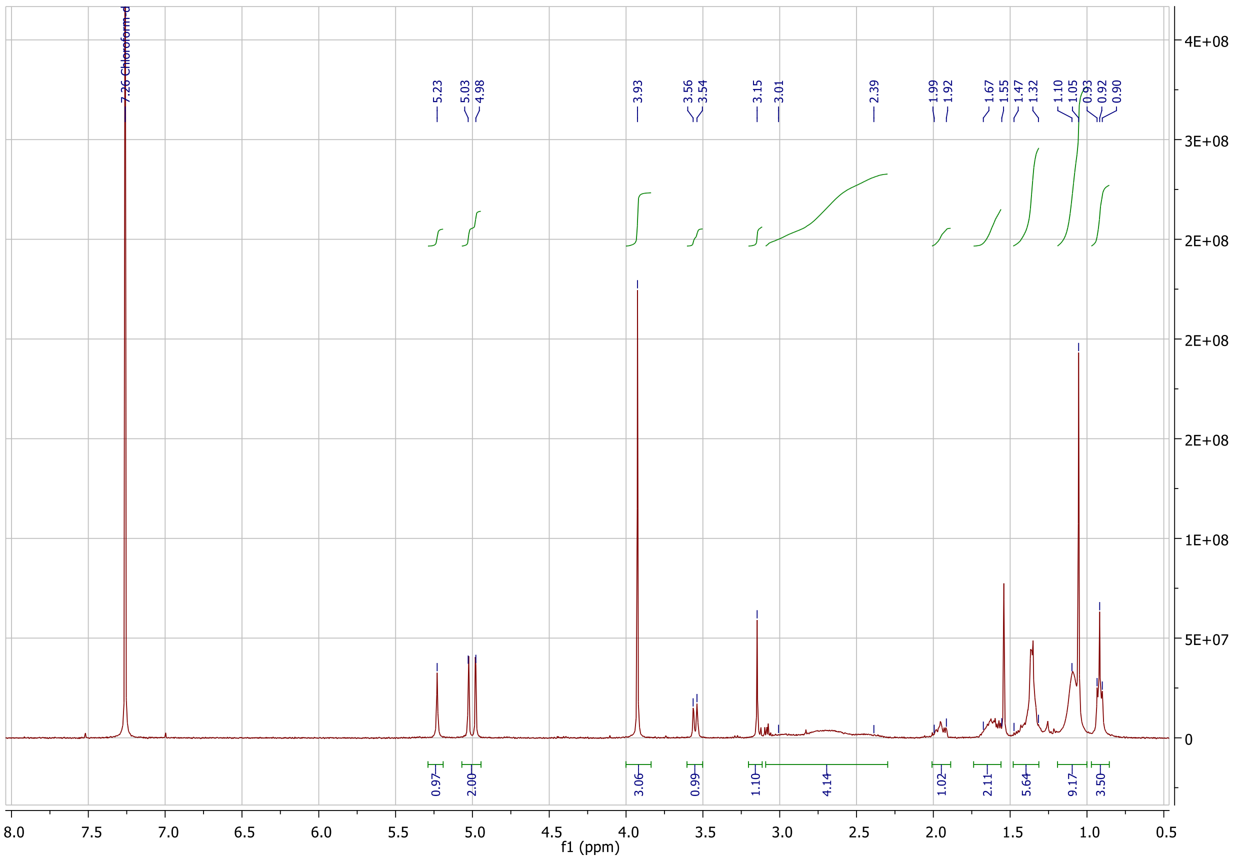

Figure S4. ^{1}H-NMR of **3**.

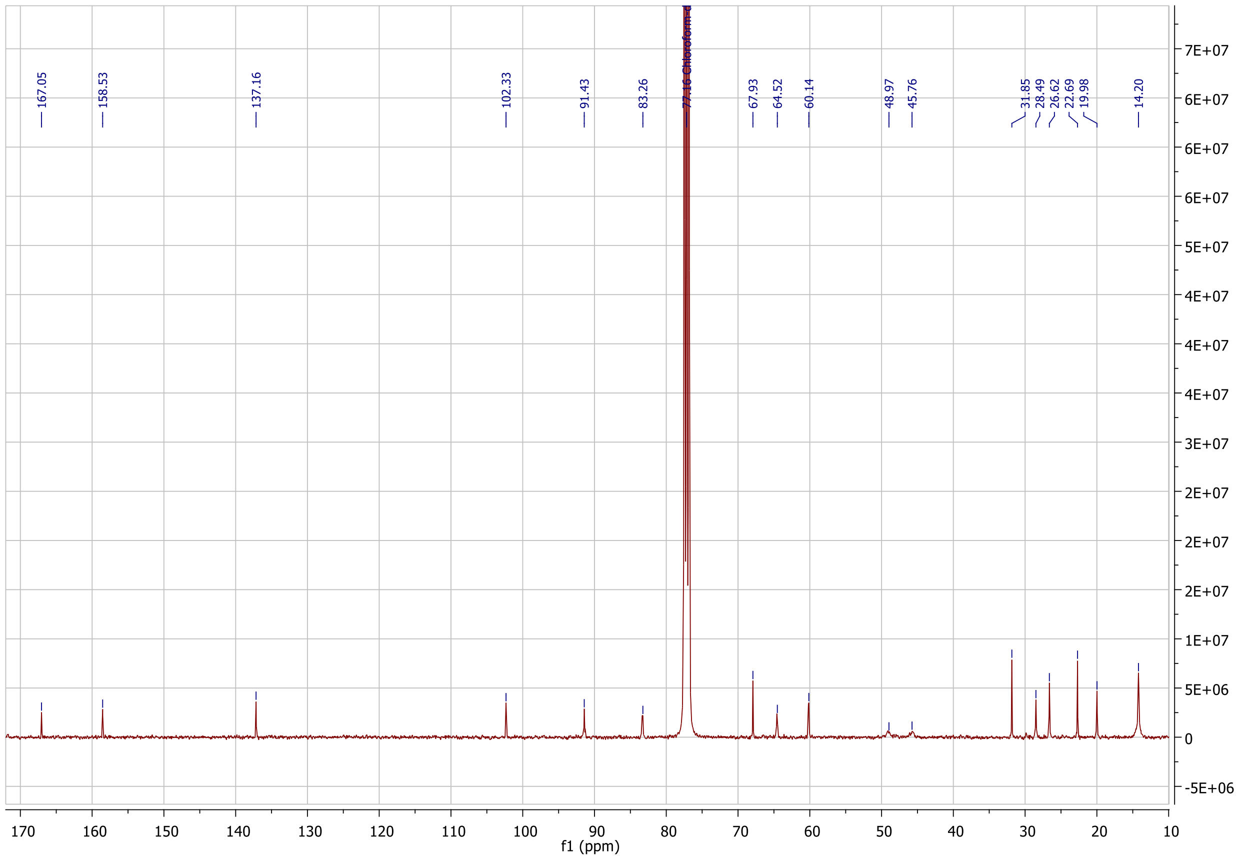

Figure S5. ^{13}C-NMR of **3**.

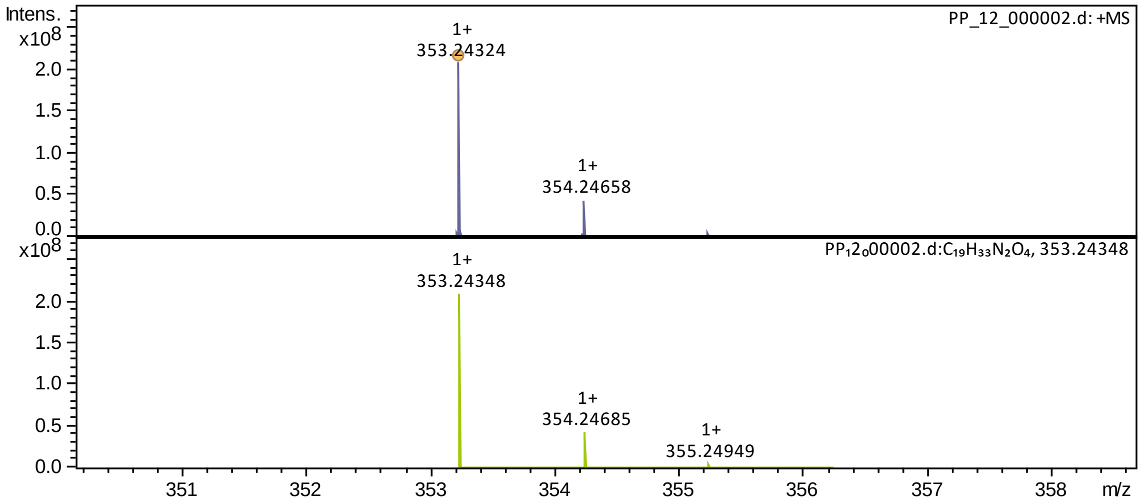

Figure S6. HRMS of **3**.

(2*S*,3*R*,4*R*)-4-[ethyl(2-hydroxyethyl)amino]-3-hydroxy-6-methoxy-3-methyl-7-methylene-2-pentyl-3,4,6,7-tetrahydropyrano[2,3-*c*]pyrrol-5(2*H*)-one (**9**)

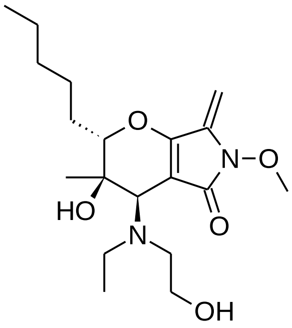

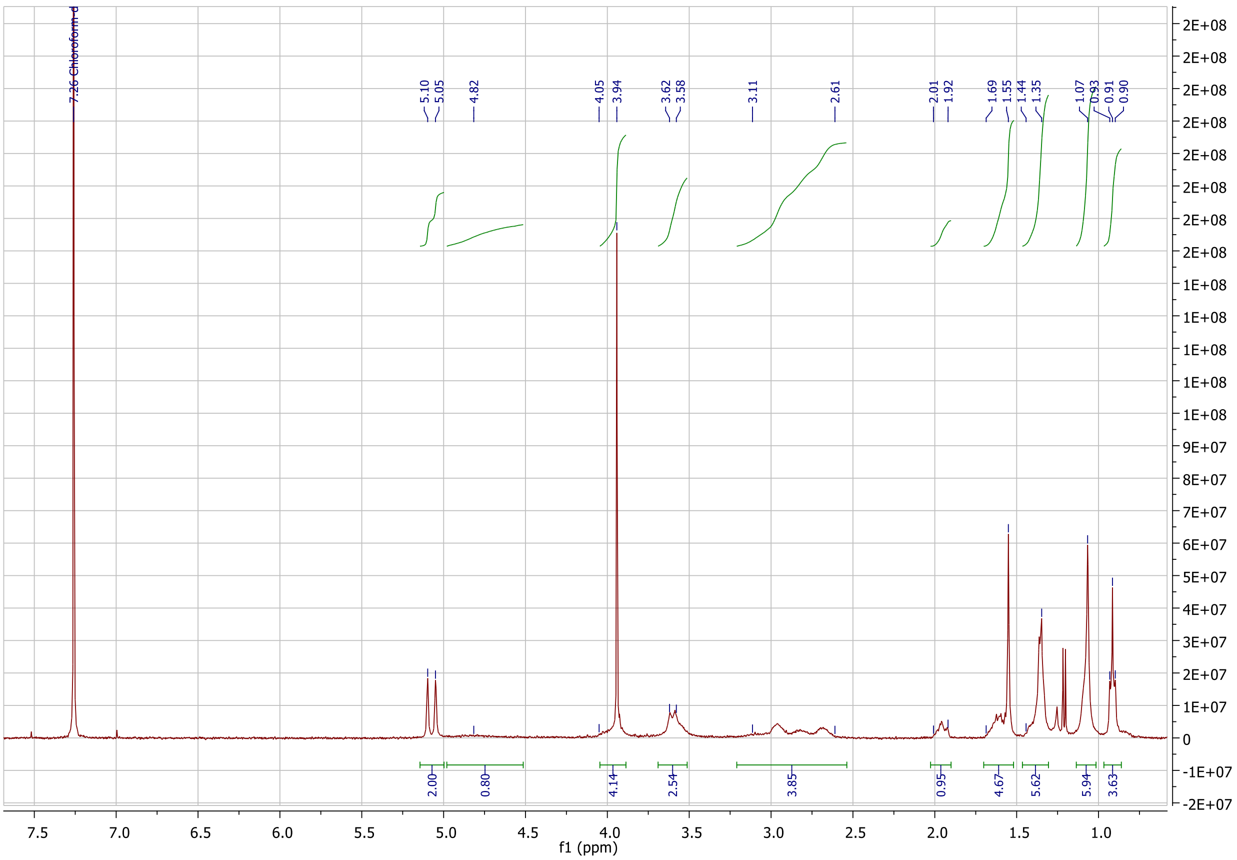

Figure S7. ^{1}H-NMR of **9**.

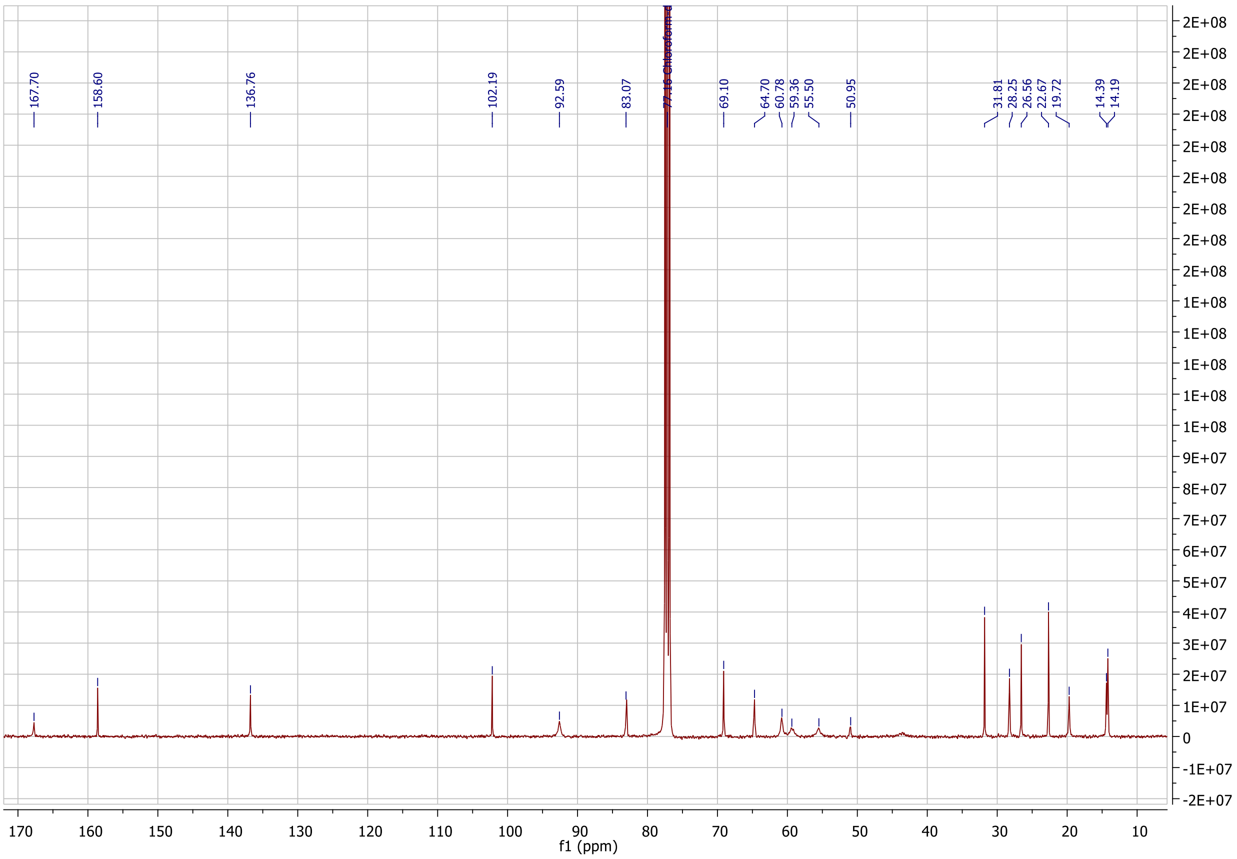

Figure S8. ^{13}C-NMR of **9**.

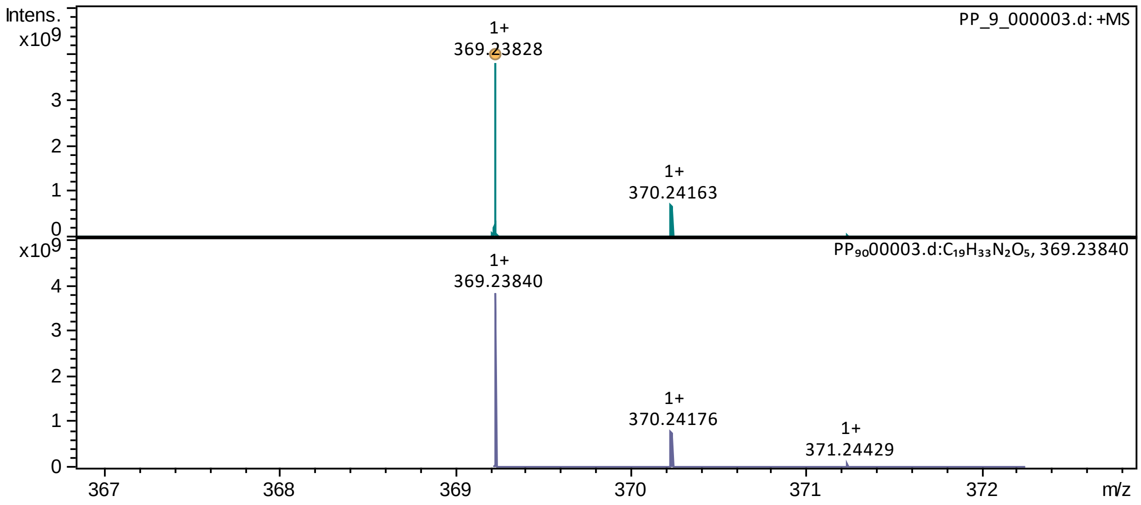

Figure S9. HRMS of **9**.

(2*S*,3*R*,4*R*)-3-hydroxy-4-[(2-hydroxyethyl)(methyl)amino]-6-methoxy-3-methyl-7-methylene-2-pentyl-3,4,6,7-tetrahydropyrano[2,3-*c*]pyrrol-5(2*H*)-one (**8**)

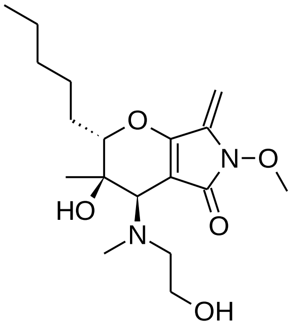

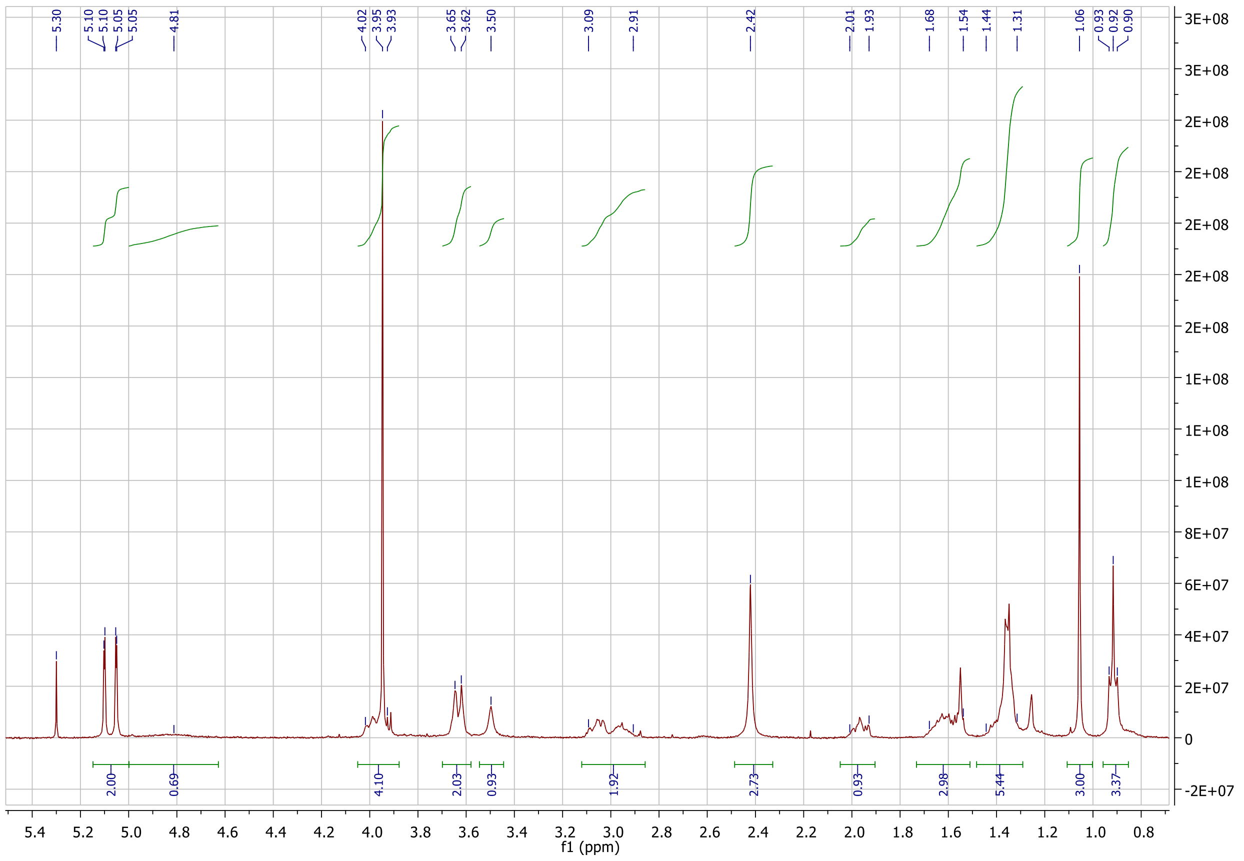

Figure S10. ^{1}H-NMR of **8**.

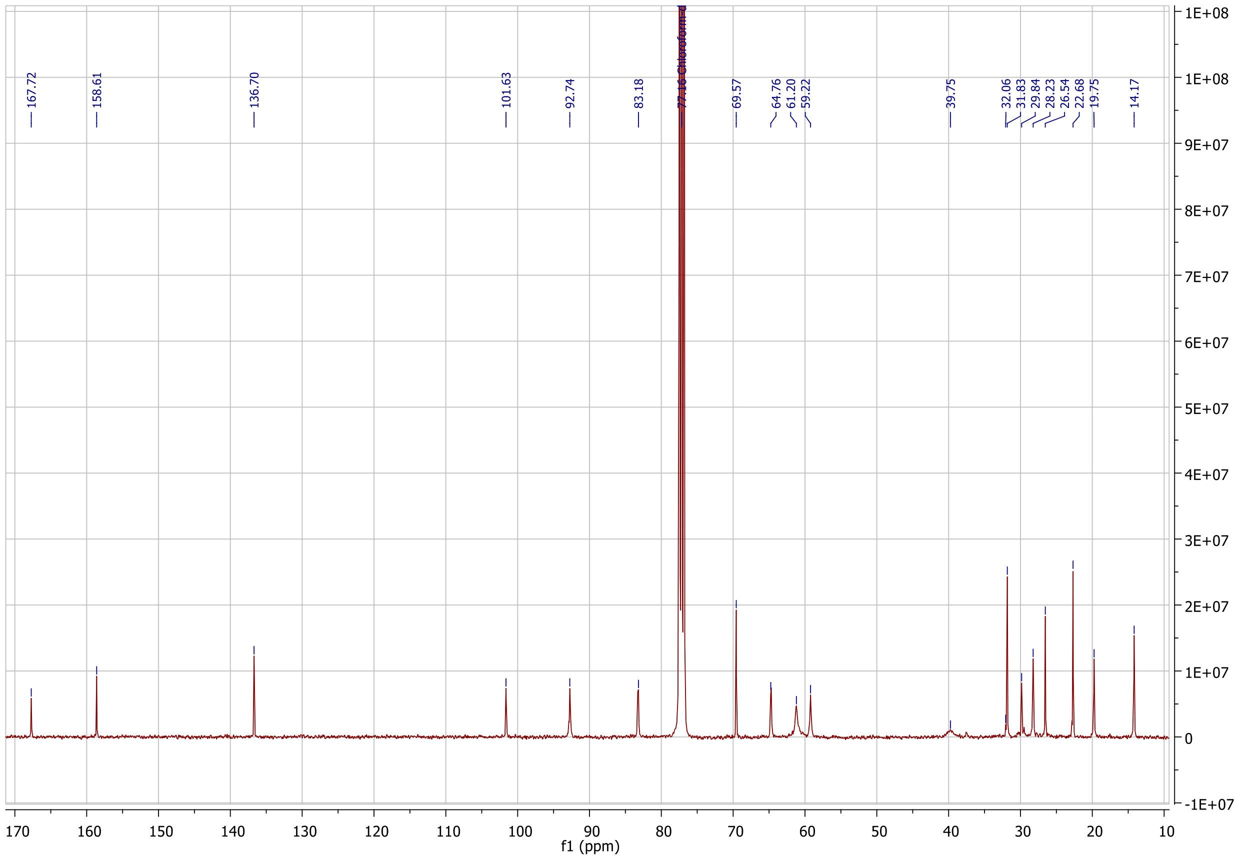

Figure S11. ^{13}C-NMR of **8**.

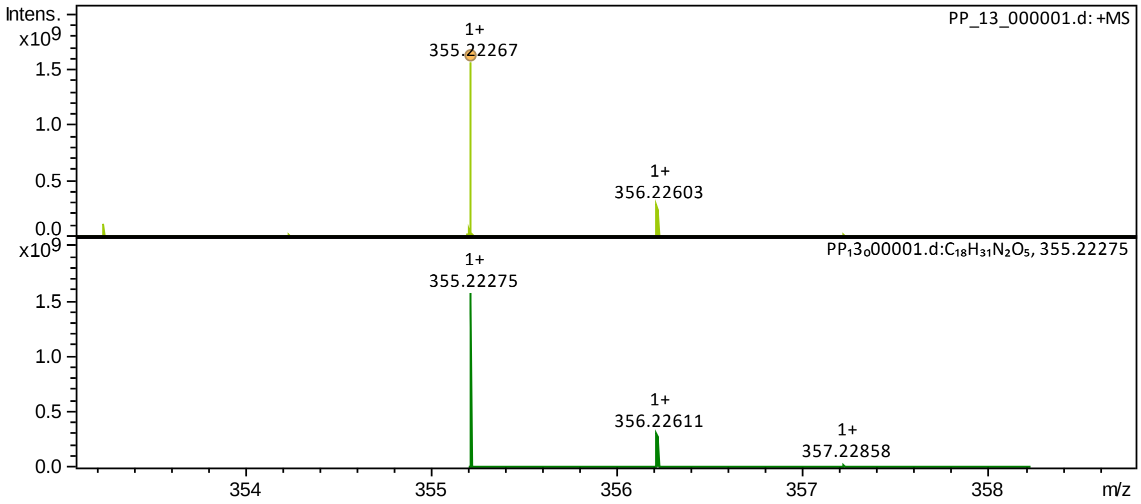

Figure S12. HRMS of **8**.

(2*S*,3*R*,4*R*)-4-(dipropylamino)-3-hydroxy-6-methoxy-3-methyl-7-methylene-2-pentyl-3,4,6,7-tetrahydropyrano[2,3-*c*]pyrrol-5(2*H*)-one (**4**)

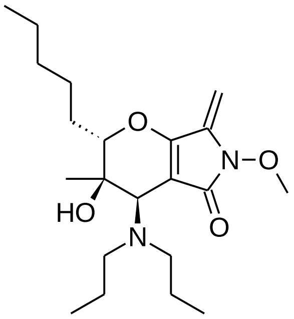

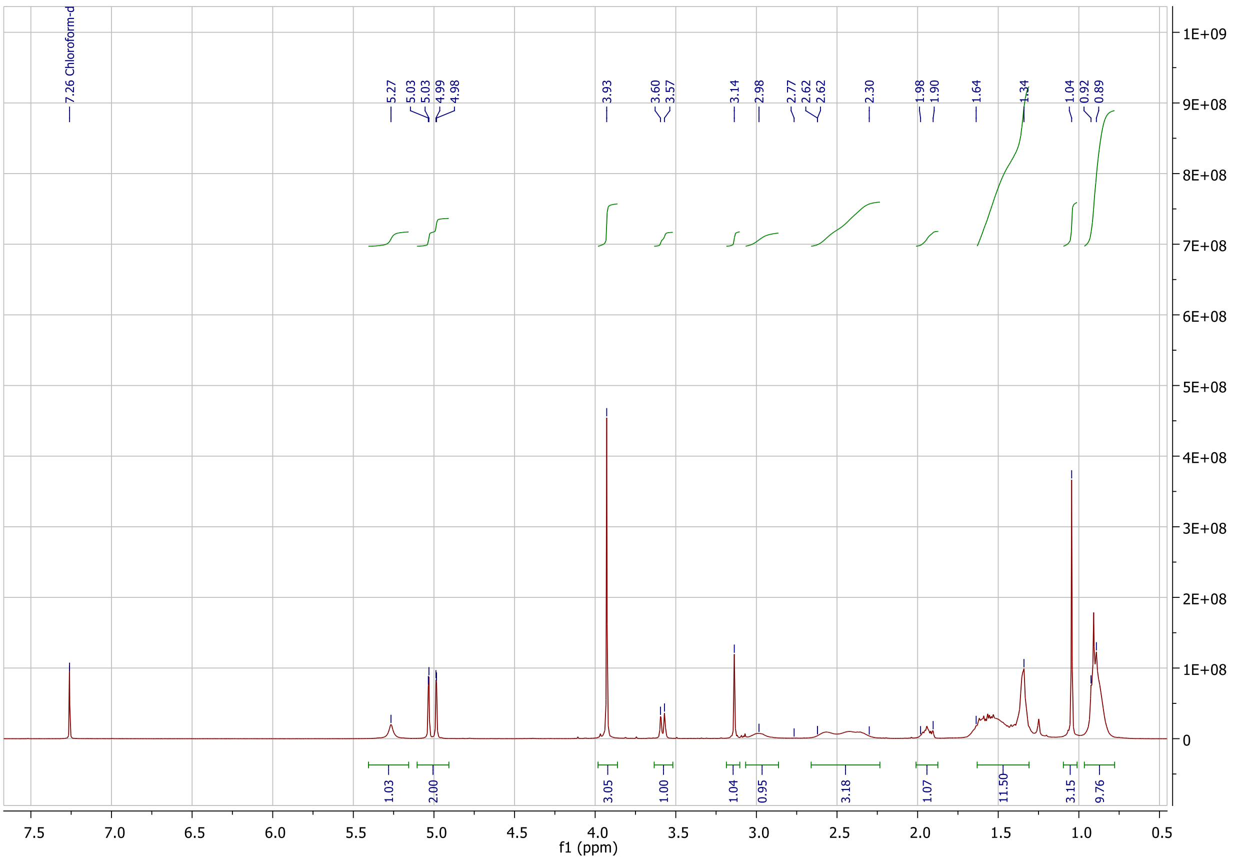

Figure S13. ^{1}H-NMR of **4**.

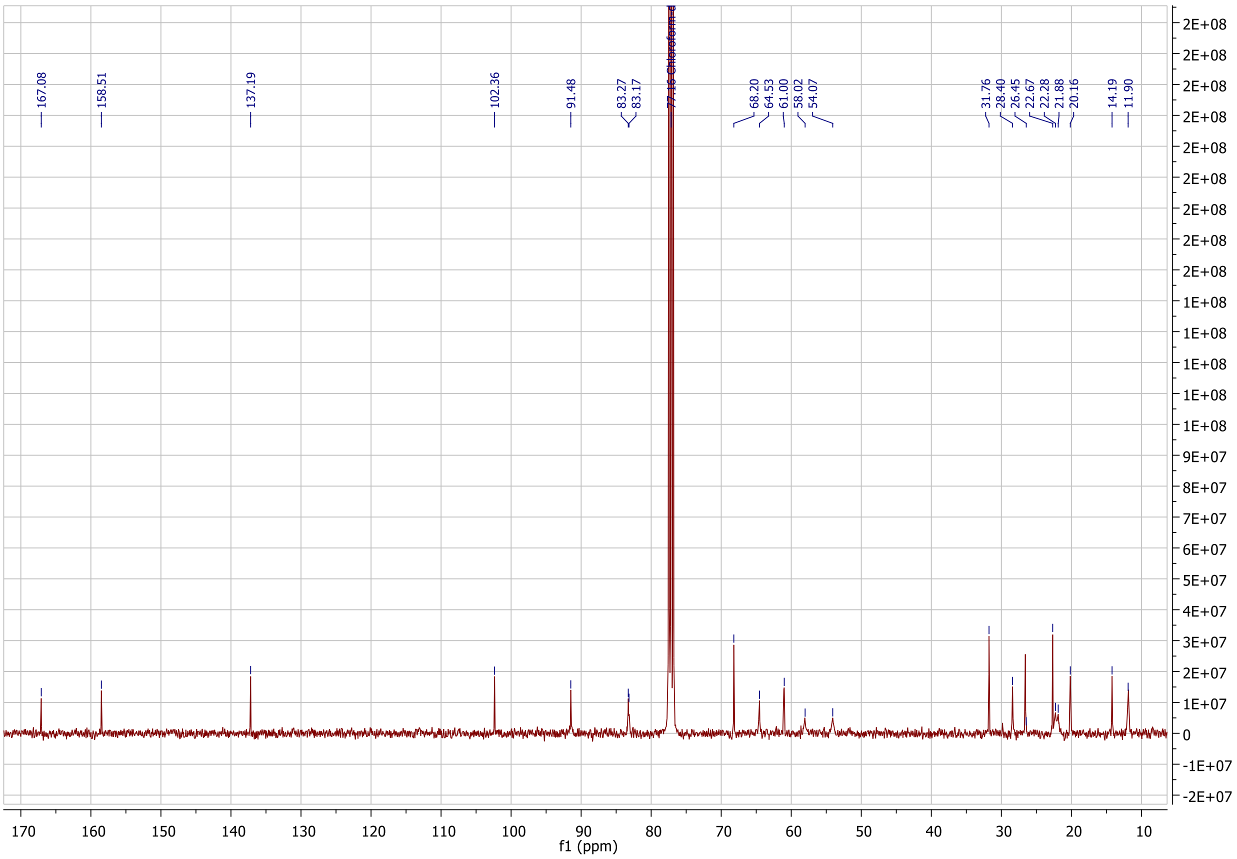

Figure S14. ^{13}C-NMR of **4**.

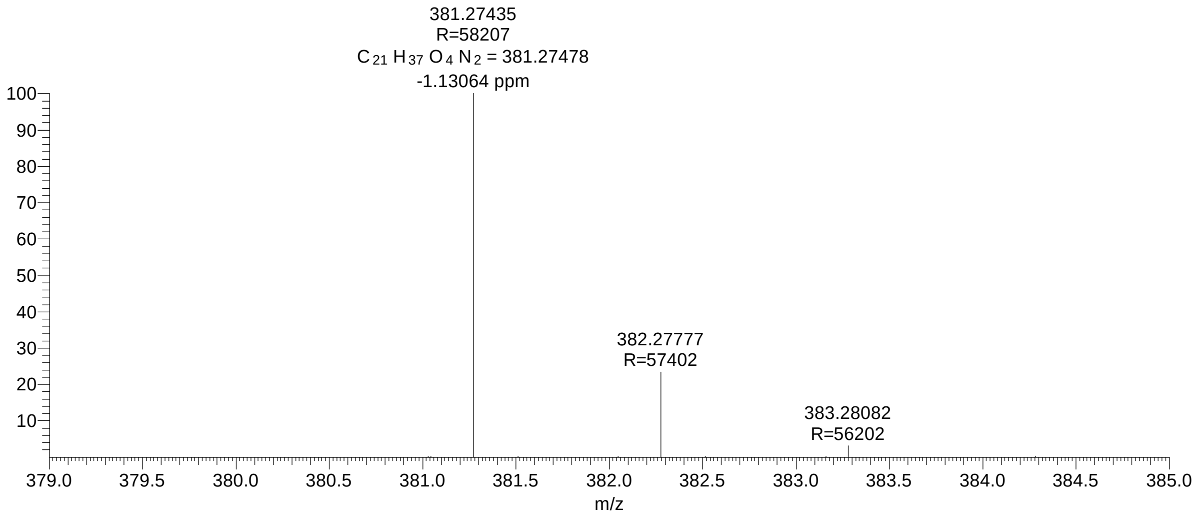

Figure S15. HRMS of **4**.

(2*S*,3*R*,4*R*)-4-(dibutylamino)-3-hydroxy-6-methoxy-3-methyl-7-methylene-2-pentyl-3,4,6,7-tetrahydropyrano[2,3-*c*]pyrrol-5(2*H*)-one (**5**)

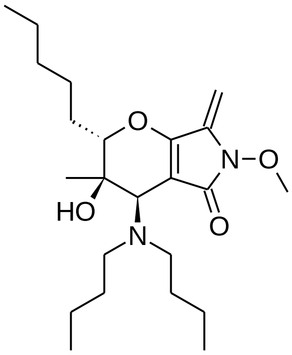

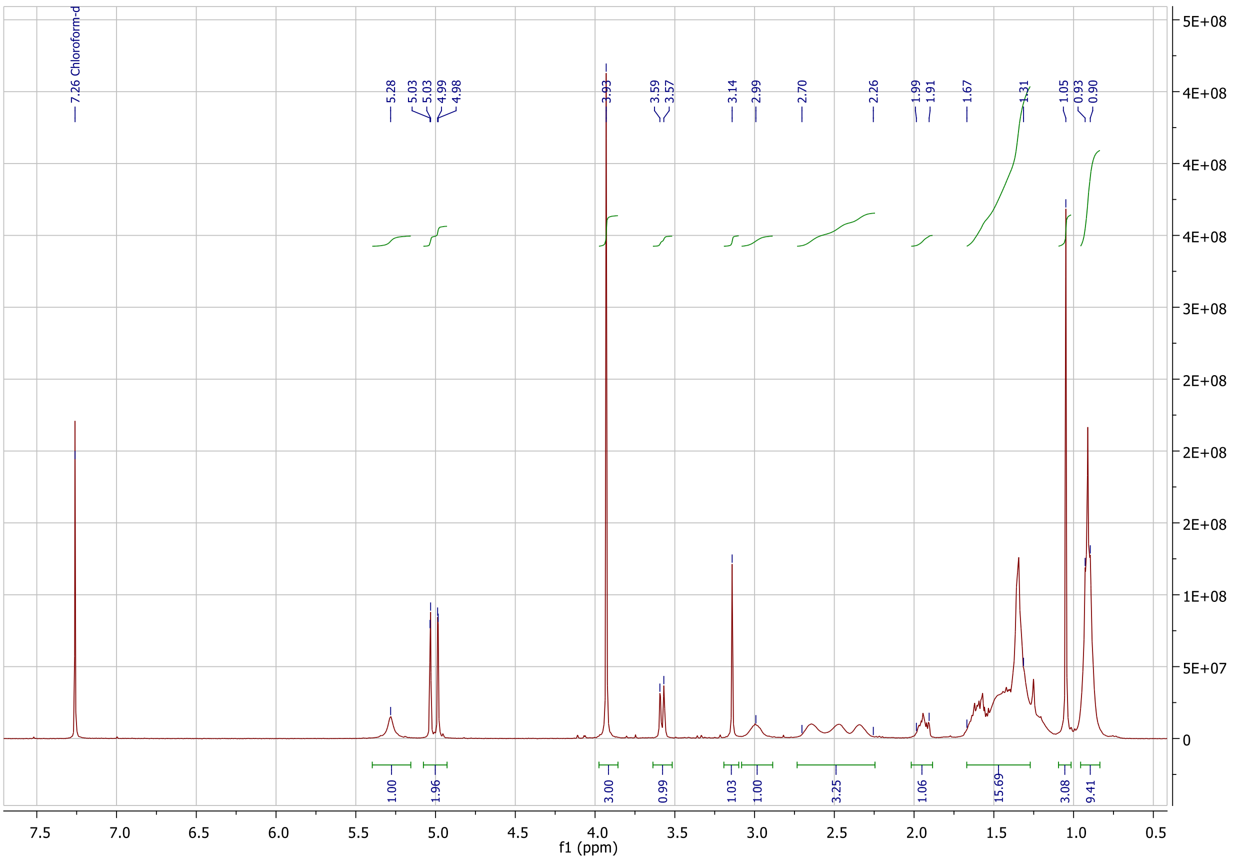

Figure S16. ^{1}H-NMR of **5**.

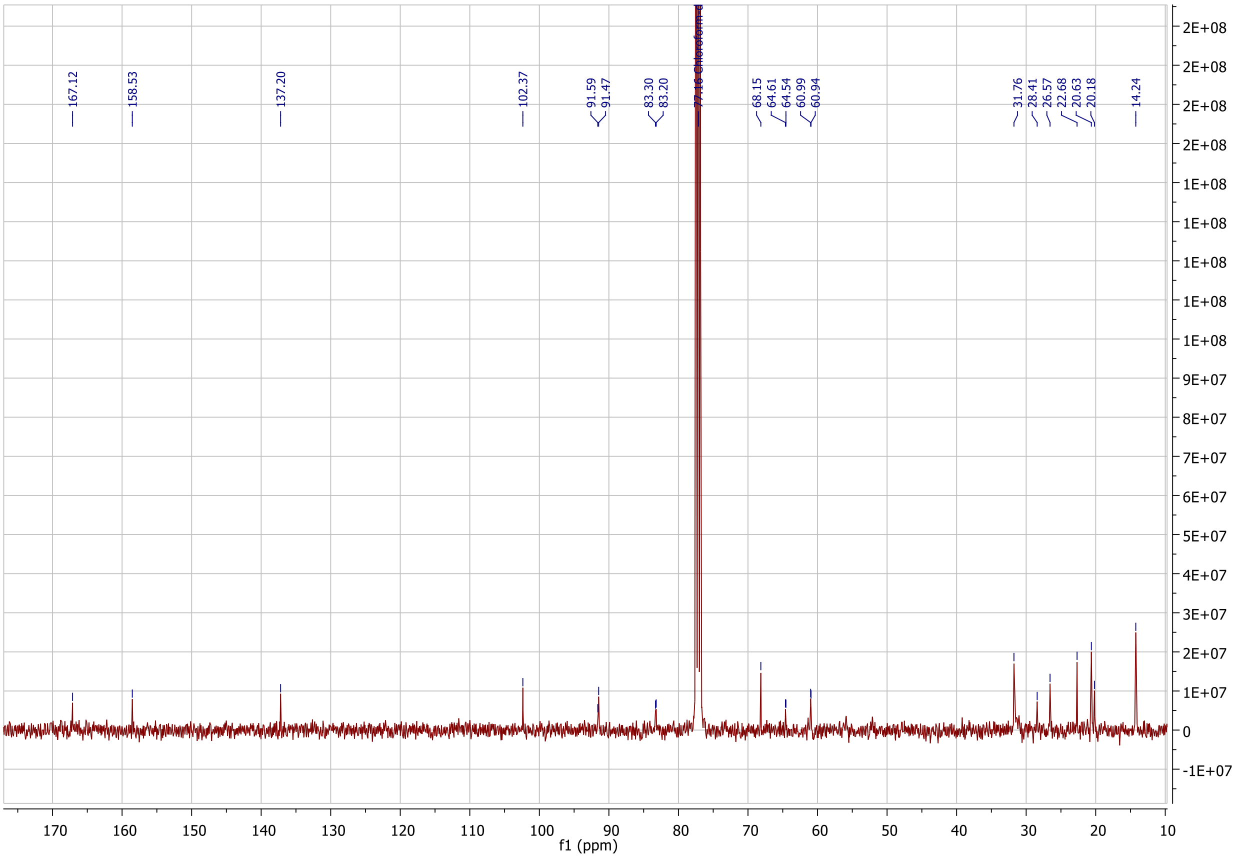

Figure S17. ^{13}C-NMR of **5**.

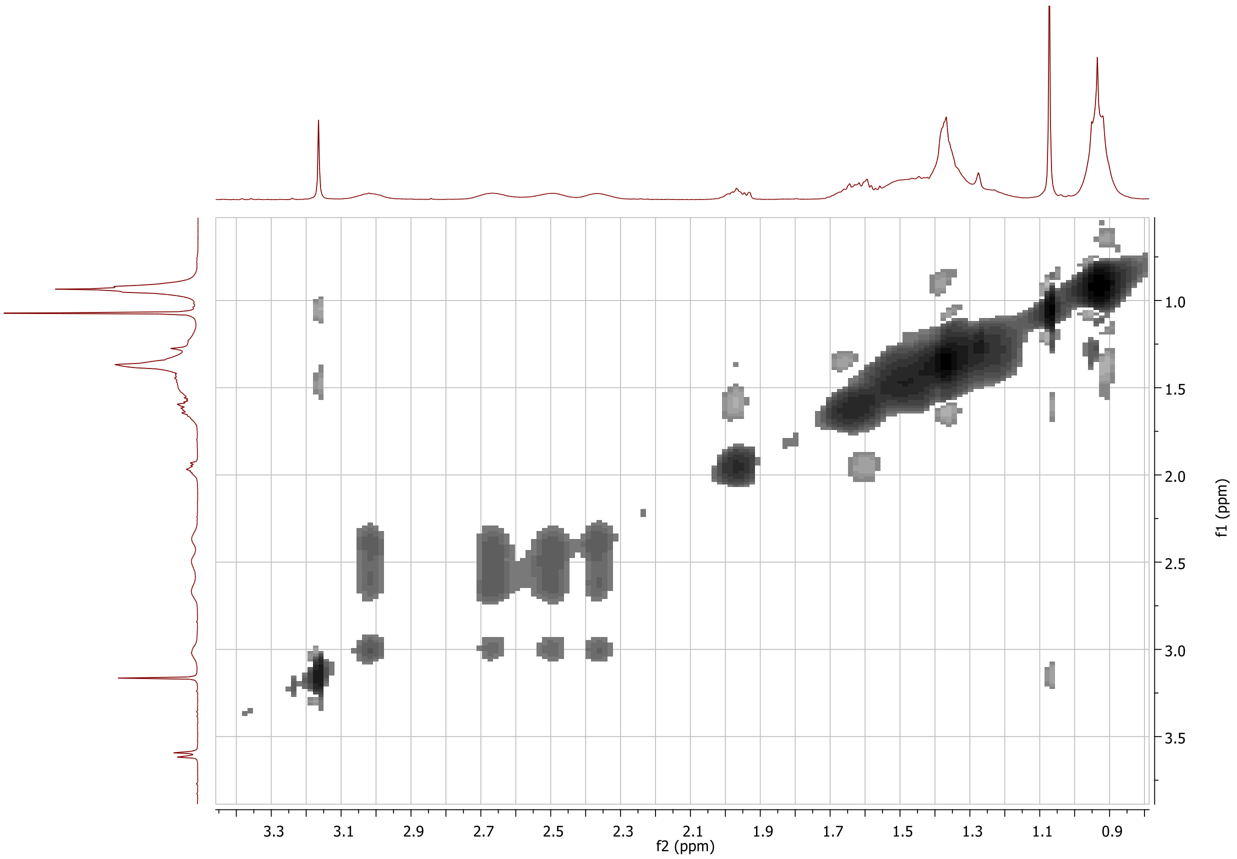

Figure S18. ^{1}H-^{1}H Roesy of **5**.

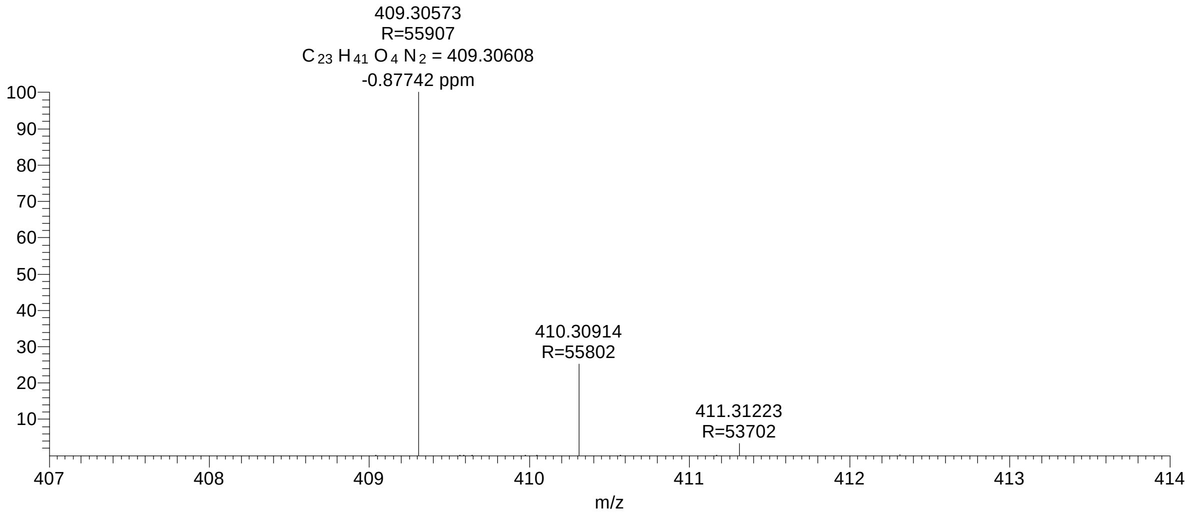

Figure S19. HRMS of **5**.

(2*S*,3*R*,4*R*)-4-(dipentylamino)-3-hydroxy-6-methoxy-3-methyl-7-methylene-2-pentyl-3,4,6,7-tetrahydropyrano[2,3-*c*]pyrrol-5(2*H*)-one (**6**)

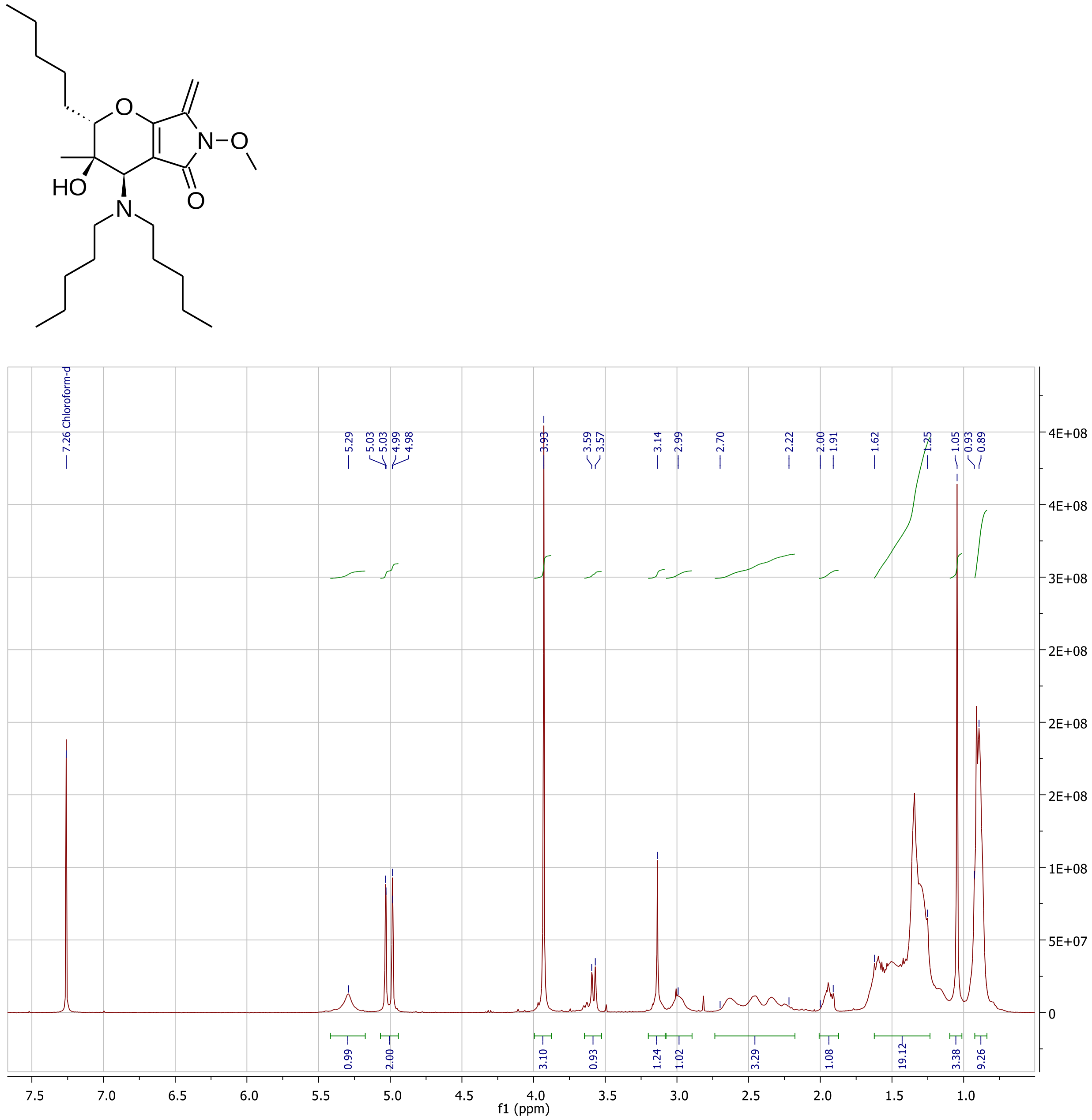

Figure S20. ^{1}H-NMR of **6**.

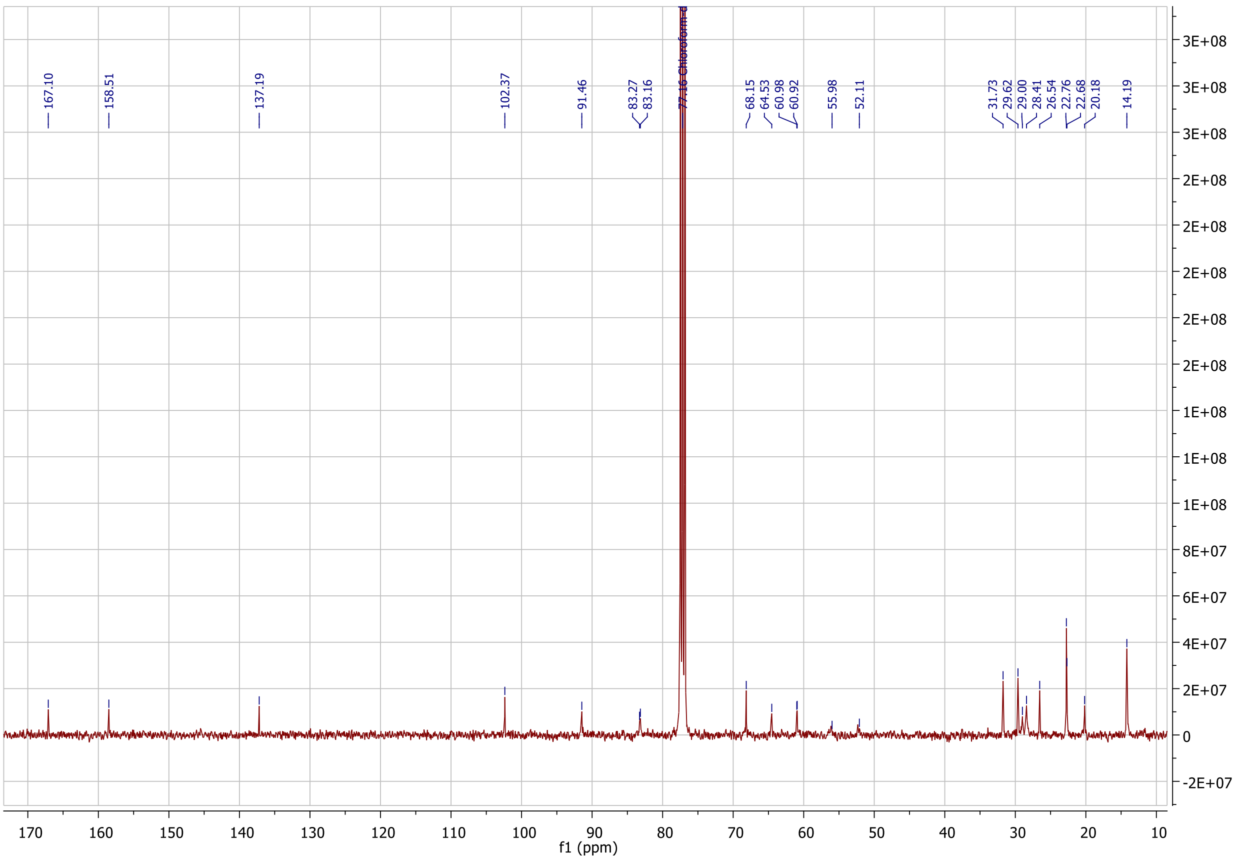

Figure S21. ^{13}C-NMR of **6**.

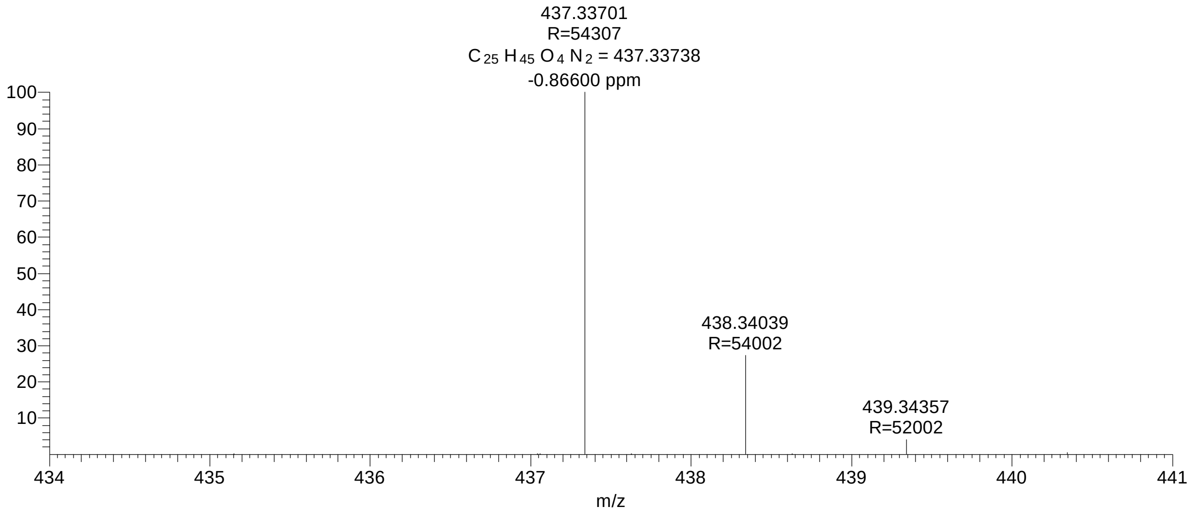

Figure S22. HRMS of **6**.

(2*S*,3*R*,4*R*)-4-(dihexylamino)-3-hydroxy-6-methoxy-3-methyl-7-methylene-2-pentyl-3,4,6,7-tetrahydropyrano[2,3-*c*]pyrrol-5(2*H*)-one (**7**)

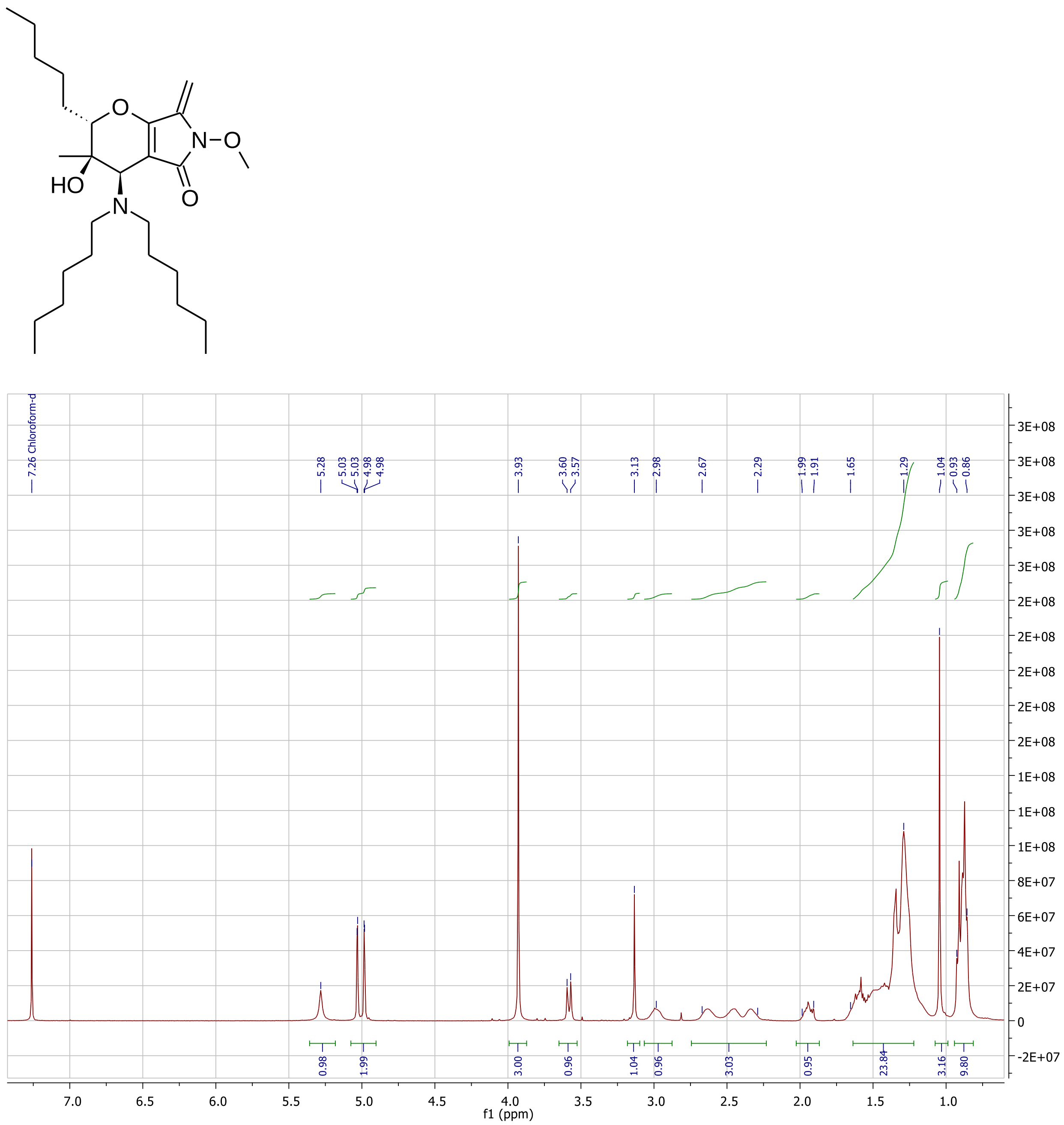

Figure S23. ^{1}H-NMR of **7**.

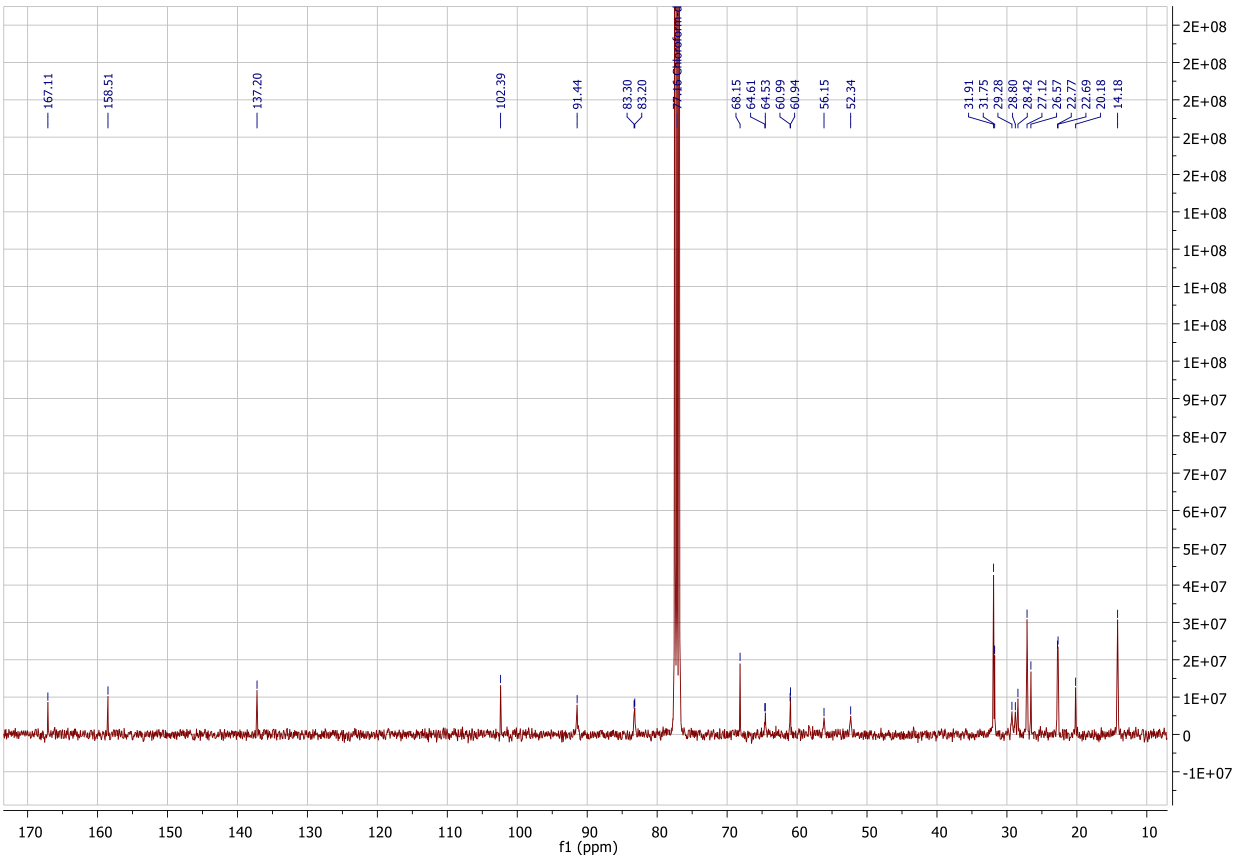

Figure S24. ^{13}C-NMR of **7**.

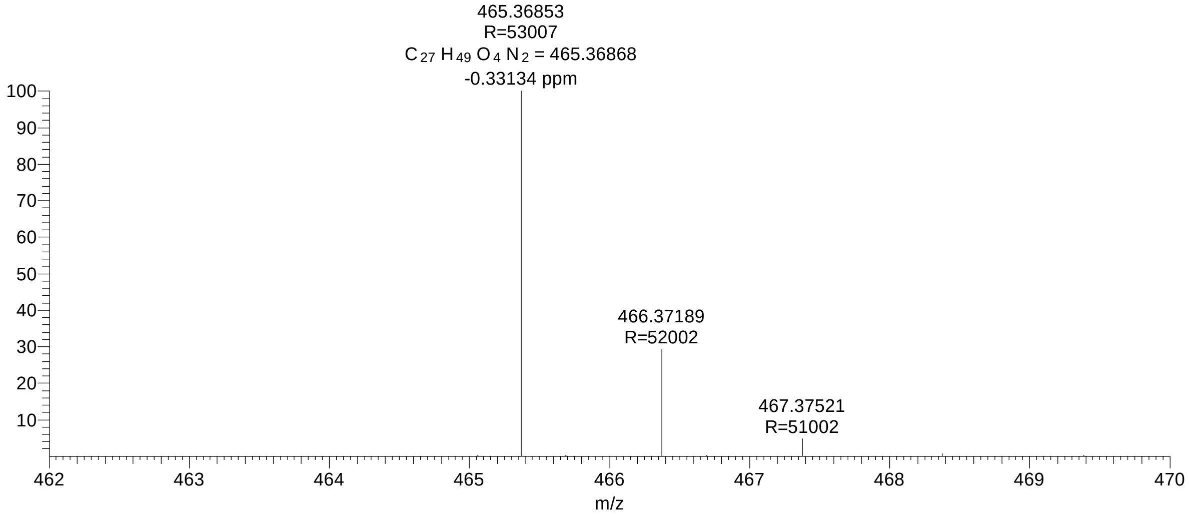

Figure S25. HRMSof**7**.

Markers of key signaling pathways

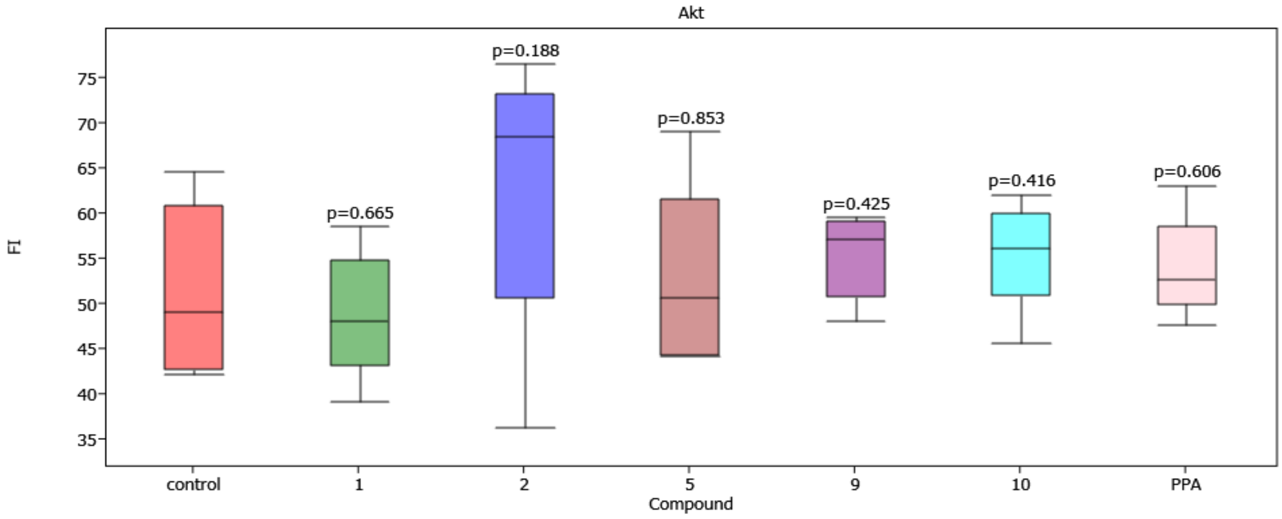

Figure S26. Box and Whisker Plot of the fluorescence intensity of Akt kinase (protein kinase B) active form. A431 epidermoid carcinoma cells were treated by 10 mkM compounds **1**, **2**, **5**, **9**, **10**, PPA for 1 hour. Cell lysates were analyzed by MILLIPLEX MAP Multi-Pathway Magnetic Bead 9-Plex kit (48-680MAG, Merck) using Bio-Rad 200 analyzer. Statistical analysis was performed using one-way ANOVA (Bio-Plex Data Pro™ Software). The differences were considered statistically significant at $p < 0.05$.

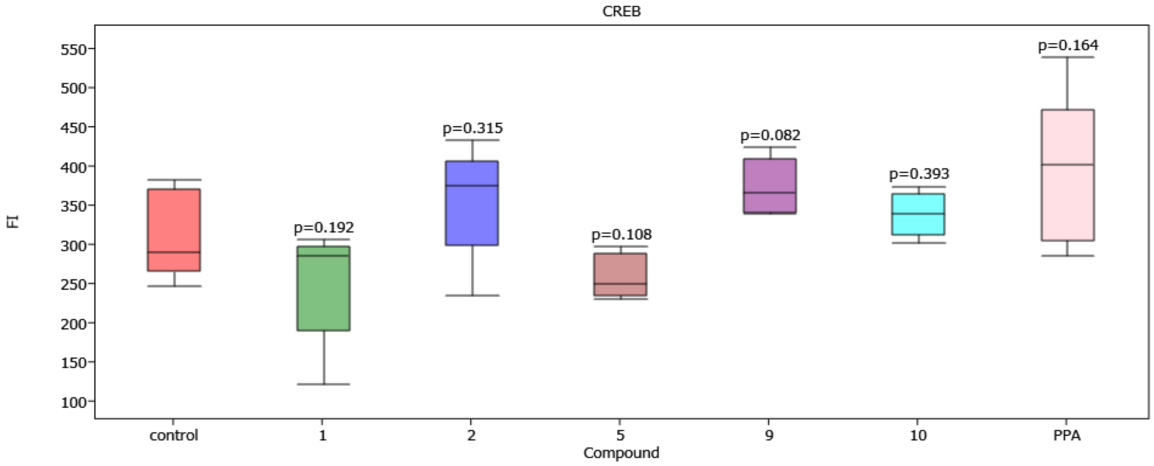

Figure S27. Box and Whisker Plot of the fluorescence intensity of CREB active form. A431 epidermoid carcinoma cells were treated by 10 mkM compounds **1**, **2**, **5**, **9**, **10**, PPA for 1 hour. Cell lysates were analyzed by MILLIPLEX MAP Multi-Pathway Magnetic Bead 9-Plex kit (48-680MAG, Merck) using Bio-Rad 200 analyzer. Statistical analysis was performed using one-way ANOVA (Bio-Plex Data Pro™ Software). The differences were considered statistically significant at $p < 0.05$.

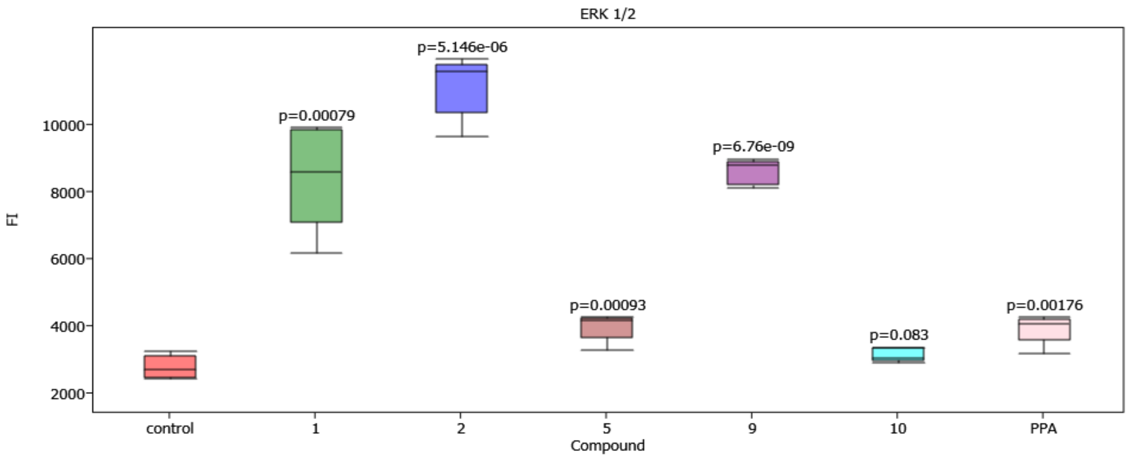

Figure S28. Box and Whisker Plot of the fluorescence intensity of ERK 1/2 (Thr185/Tyr187) active form. A431 epidermoid carcinoma cells were treated by 10 mkM compounds **1**, **2**, **5**, **9**, **10**, PPA for 1 hour. Cell lysates were analyzed by MILLIPLEX MAP Multi-Pathway Magnetic Bead 9-Plex kit (48-680MAG, Merck) using Bio-Rad 200 analyzer. Statistical analysis was performed using one-way ANOVA (Bio-Plex Data Pro™ Software). The differences were considered statistically significant at $p < 0.05$.

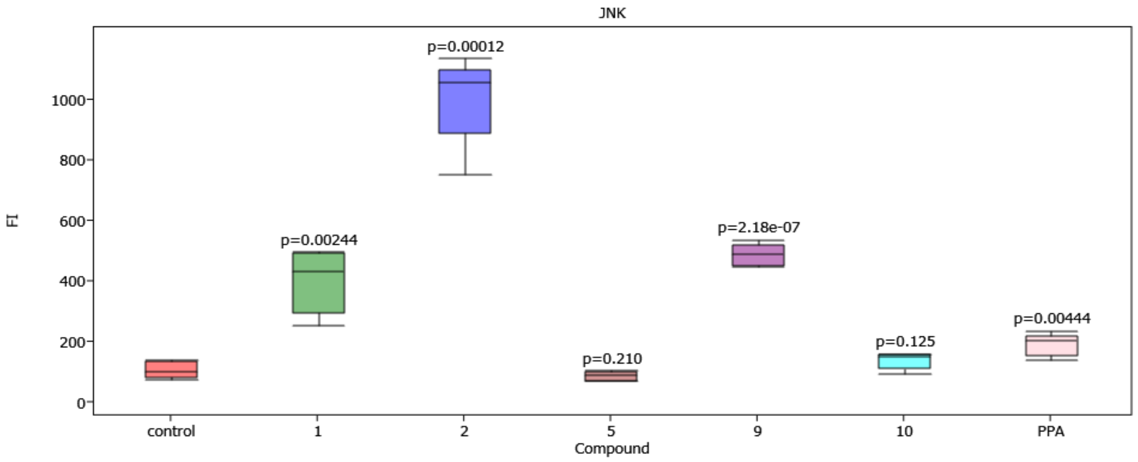

Figure S29. Box and Whisker Plot of the fluorescence intensity of JNK kinaze active form. A431 epidermoid carcinoma cells were treated by 10 mkM compounds **1**, **2**, **5**, **9**, **10**, PPA for 1 hour. Cell lysates were analyzed by MILLIPLEX MAP Multi-Pathway Magnetic Bead 9-Plex kit (48-680MAG, Merck) using Bio-Rad 200 analyzer. Statistical analysis was performed using one-way ANOVA (Bio-Plex Data Pro™ Software). The differences were considered statistically significant at $p < 0.05$.

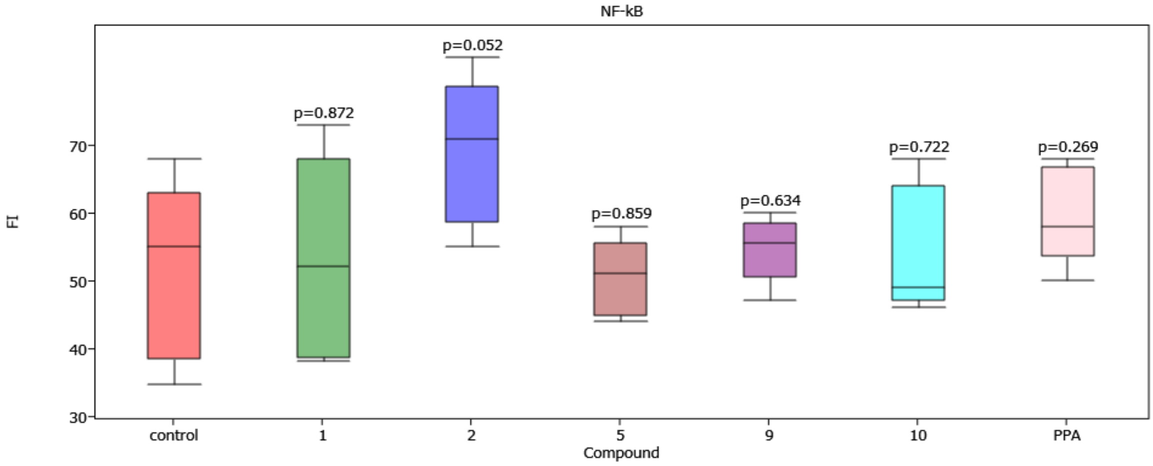

Figure S30. Box and Whisker Plot of the fluorescence intensity of NF-κB active form. A431 epidermoid carcinoma cells were treated by 10 mkM compounds **1**, **2**, **5**, **9**, **10**, PPA for 1 hour. Cell lysates were analyzed by MILLIPLEX MAP Multi-Pathway Magnetic Bead 9-Plex kit (48-680MAG, Merck) using Bio-Rad 200 analyzer. Statistical analysis was performed using one-way ANOVA (Bio-Plex Data Pro™ Software). The differences were considered statistically significant at $p < 0.05$.

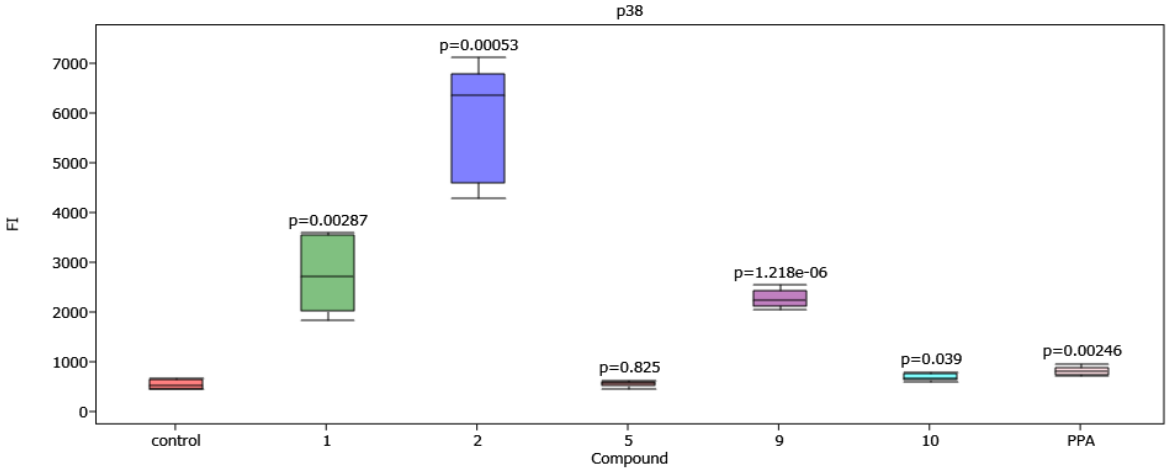

Figure S31. Box and Whisker Plot of the fluorescence intensity of protein kinase p38 active form. A431 epidermoid carcinoma cells were treated by 10 mkM compounds **1**, **2**, **5**, **9**, **10**, PPA for 1 hour. Cell lysates were analyzed by MILLIPLEX MAP Multi-Pathway Magnetic Bead 9-Plex kit (48-680MAG, Merck) using Bio-Rad 200 analyzer. Statistical analysis was performed using one-way ANOVA (Bio-Plex Data Pro™ Software). The differences were considered statistically significant at $p < 0.05$.

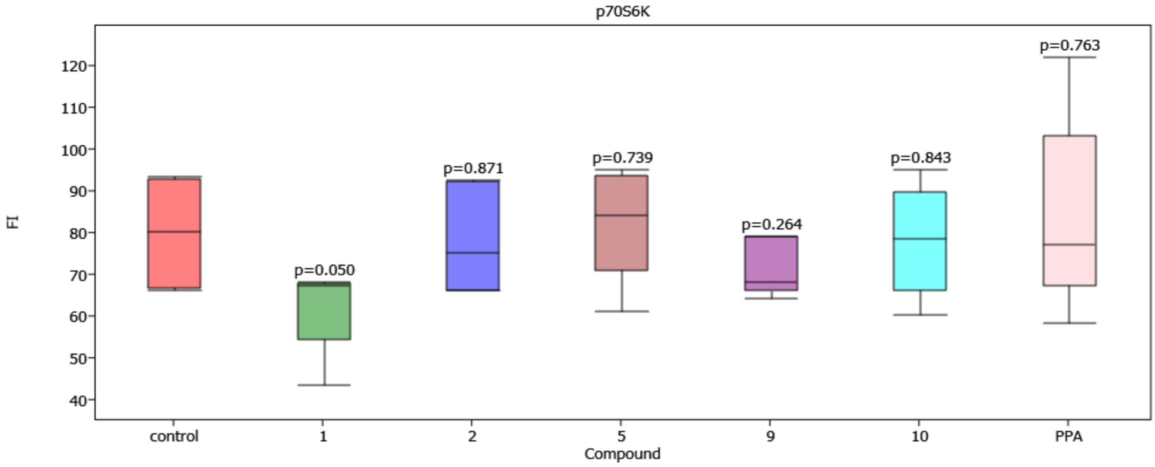

Figure S32. Box and Whisker Plot of the fluorescence intensity of protein kinase S6 бета-1(p70S6K) active form. A431 epidermoid carcinoma cells were treated by 10 mkM compounds **1**, **2**, **5**, **9**, **10**, PPA for 1 hour. Cell lysates were analyzed by MILLIPLEX MAP Multi-Pathway Magnetic Bead 9-Plex kit (48-680MAG, Merck) using Bio-Rad 200 analyzer. Statistical analysis was performed using one-way ANOVA (Bio-Plex Data Pro™ Software). The differences were considered statistically significant at $p < 0.05$.

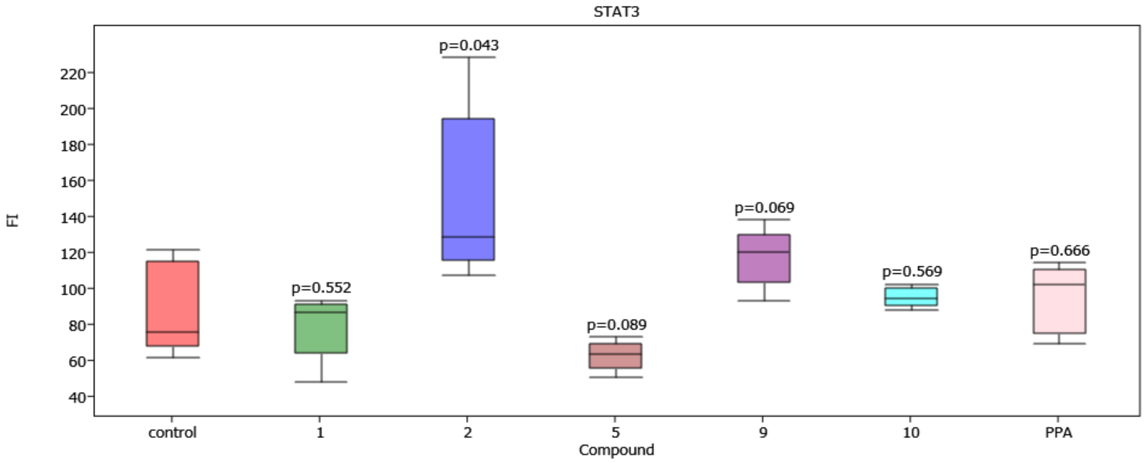

Figure S33. Figure 32. Box and Whisker Plot of the fluorescence intensity of STAT3 active form. A431 epidermoid carcinoma cells were treated by 10 mkM compounds **1**, **2**, **5**, **9**, **10**, PPA for 1 hour. Cell lysates were analyzed by MILLIPLEX MAP Multi-Pathway Magnetic Bead 9-Plex kit (48-680MAG, Merck) using Bio-Rad 200 analyzer. Statistical analysis was performed using one-way ANOVA (Bio-Plex Data Pro™ Software). The differences were considered statistically significant at $p < 0.05$.

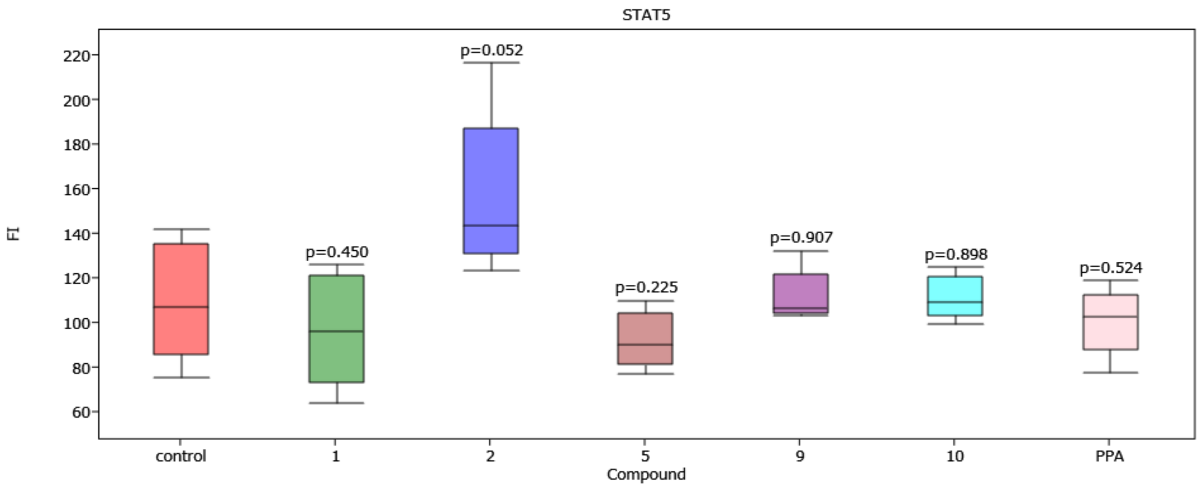

Figure S34. Figure 32. Box and Whisker Plot of the fluorescence intensity of STAT5 active form. A431 epidermoid carcinoma cells were treated by 10 mkM compounds **1**, **2**, **5**, **9**, **10**, PPA for 1 hour. Cell lysates were analyzed by MILLIPLEX MAP Multi-Pathway Magnetic Bead 9-Plex kit (48-680MAG, Merck) using Bio-Rad 200 analyzer. Statistical analysis was performed using one-way ANOVA (Bio-Plex Data Pro™ Software). The differences were considered statistically significant at $p < 0.05$.
